# Detection of potential biodeterioration risks for tempera painting in 16th century exhibits from State Tretyakov Gallery

**DOI:** 10.1371/journal.pone.0230591

**Published:** 2020-04-02

**Authors:** Alexander Zhgun, Darya Avdanina, Kirill Shumikhin, Nikolay Simonenko, Elena Lyubavskaya, Ivan Volkov, Victor Ivanov

**Affiliations:** 1 Research Center of Biotechnology RAS, Moscow, Russia; 2 State Tretyakov Gallery, Moscow, Russia; 3 Kurnakov Institute of General and Inorganic Chemistry, RAS, Moscow, Russia; 4 Institute of Physics and Technology, Dolgoprudniy, Russia; USDA Forest Service, UNITED STATES

## Abstract

In this study, we investigated biodeterioration of materials used in tempera painting by analyzing the structure of the microbiome in ancient tempera paintings exhibited in State Tretyakov Gallery, Moscow, Russia. Samples were obtained from 16^th^-century paintings, including a grand Russian Orthodox icon “The Church Militant” (all exhibits were without visible signs of biodeterioration), and from surrounding walls and ceilings (with vast zones of visible microbial growth). A number of microorganisms isolated from visible signs of environmental bio-damage were also detected in tempera paintings kept in temperature- and humidity-controlled conditions unfavorable for the growth of microflora. To determine the biodegrading potential of the microbiome for tempera paintings, we developed a set of mock layers from paintwork materials used in tempera painting of 16^th^ century and their modern analogues and inoculated them with cultures containing filamentous fungi and bacteria. The susceptibility to microbial degradation of individual tempera painting materials was examined by micro-Fourier Transform Infrared (FTIR) spectroscopy, which enabled detection of even invisible signs of biodeterioration. The results indicate that the microorganisms isolated from paintings and surrounding areas in the museum are capable of causing significant damage of various tempera materials, among which varnishes were the most resistant; however, the addition of antiseptic (sodium pentachlorophenolate) can inhibit microbial growth on sturgeon glue.

## Introduction

Microbiological community play an essential role in the destruction of cultural heritage [1–3]. Microorganisms from various systematic groups that can damage works of art, such as tempera painting or oil painting on canvas, have been extensively studied in recent years [4]. The primary factors in the biodeterioration of objects belonging to cultural heritage are environmental conditions and chemical composition [5]. From the 18^th^ century, it has been well known that paintings exposed to high humidity may be colonized by mold [6]. Ancient Russian tempera paintings (ARTPs), including its most valuable specimens–icons (easel tempera painting) and tempera-painted statues, contain a number of organic and inorganic components which can be used by microorganisms for growth. In icon manufacturing, one or several wooden boards made of various types of trees such as linden, pine, maple, cypress, and spruce were used as a basis for ARTPs; the stitches were glued to canvas (also called **pavoloka**) soaked in sturgeon, hide, or bone glue. Then, the ground layer (**levkas**) made of chalk or hydrated lime mixed with sturgeon or other animal glue was put and the drawing was made on levkas. For icon painting, egg and casein tempera were used; in case of egg tempera, organic and inorganic pigments were ground in egg yolk or white [7,8]. Furthermore, various plasticizers such as glycerin, honey, or linseed oil were applied to provide the elasticity of paints, whereas binders such as gum arabic were used to disperse watercolor pigments and waxes, and varnishes of different composition and rosin were used to increase the optical stability and protect tempera paints [9]. Most of these materials can potentially support the growth of various paint-deteriorating microorganisms. In addition, prior to transfer from the original locations (churches, monasteries and other places) to museum funds that provide stable storage conditions, the ARTPs are subjected to the influence of occasional damaging factors favoring microbial growth, such as accumulation of surface dirt, dust, and volatile hydrocarbons [10] or dead and live cells [11].

Art museums are usually located in historical buildings, which by themselves have a significant architectural and cultural value. The historical building of the State Tretyakov Gallery (STG) was used as a repository of cultural objects, especially paintings, since the middle of the 19^th^ century. During this time, the environment of the gallery, including extensive visiting, specific storage conditions, and exposure to old materials belonging to the epoch of its construction, could have supported the evolvement of a very specific microbial community [12]. Thus, regulated storage conditions created by strict maintenance of temperature (19°C) and humidity (55%) levels could, on the one hand, suppress the growth of conventional microbiota, but on the other hand, serve as a selection tool favoring survival of xerophilic and sporulating microorganisms. Such kind of microbiota can resist extremely low moisture content and endure unfavorable conditions for a long time. For example, it was recently demonstrated that a unique microbiome, including xerophilic fungi, could be formed in a library depository [13]. Therefore, any deviation from the standard storage conditions may induce microbial growth in the museum and the most urgent task in the conservation of art objects is timely identification of microbial infection, which should be constantly monitored in order to prevent irreversible damage of art objects.

One of the dynamically developing approaches to this problem is spectral analysis, which provides detection of a wide range of compounds in the objects of cultural heritage, in particular oil and tempera paintings. Fourier Transform Infrared (FTIR) spectroscopy, together with Raman spectroscopy, makes it possible to determine, with sufficient accuracy, the type of binder [14] or the array of pigments used [15]. Infrared spectroscopy has been shown to be a sensitive method for identifying biological contamination responsible for degradation of wood and mural paintings [16,17], indicating its utility for monitoring microbial colonization of art objects. In this regard, special attention is paid to the detection of fungal infection. Sensitive diagnostics would promote the development of effective preventive antiseptic materials that would suppress microbial growth with a minimal destructive effect on the art object [1,5,6]. Traditional antiseptics used in tempera paintings have limitations [6] and there is an urgent need to develop novel modern antiseptics with broad-spectrum antimicrobial activity and high efficacy, which at the same time would be safe for paintings and museum staff.

The main aim of this study was to estimate the potential risk of microbial colonization for tempera paintings of 16^th^ century, stored for a long time in STG halls with signs of biodegradation. To achieve it, we characterized and isolated microorganisms from the surfaces of tempera exhibits (without visible signs of biodeterioration) and from visible biological lesions on the walls, determined the biodegradation potential of cultivated isolates against mock layers created with various materials used in tempera painting, and assessed the microbial growth and surface destruction using micro-FTIR spectroscopy.

## Materials and methods

### Sampling

Samples were obtained from February 19 to 27, 2018, in Paintings of Ancient Rus Halls № 56, 57 and 61 in the main historical building of STG (Lavrushinsky per. 10, Moscow, Russia). Sixty six samples were collected from tempera paintings dated around 16^th^ century and belonging to cultural heritage: grand Russian Orthodox icon “The Church Militant” (**object I**), tempera-covered limestone bust of “Saint George the Victorious” (**object II**), and Russian Orthodox icon “Great Martyr St. Demetrius of Thessalonica” (**object III**). Description of these exhibits, including used materials, is provided in Materials and Methods 1.1 in S1 File. After emergency transfer of exhibits and dismantling of the frontal wall in Hall № 61, samples were also taken from various areas of panels, inner walls, ceiling, cornices, and baseboards. All samples were collected with the permission of the Chief Guardian of STG objects Tatyana Gorodkova and under surveillance of the curator from the department of ancient Russian art Valentina Uhanova; sampling sites in Hall № 61 are shown in S1 and S2 Figs.

Surface samples were taken from the area of 2 cm^2^ by non-invasive procedure using dry sterile cotton buds, which were then placed in transport Maximum Recovery Diluent (MRD) medium (Merck, Germany) and shaken for 1 h at 24°C. Then, the swabs were aseptically pressed and removed, and 100 μL of the remaining suspensions (designated initial samples) were used to inoculate slant agar media: Lysogeny Broth (LB: 10 g/L Tryptone, 5 g/L Yeast extract, 10 g/L NaCl, 20 g/L agar) to assess bacterial growth and Czapek-Dox (CD) (30 g/L sucrose, 2 g/L NaNO_3_, 1 g/L K_2_HPO4, 0.5 g/L MgSO_4_×7 H _2_O, 0.5 g/L KCl, 0.01 g/L FeSO_4_×7 H_2_O, 20 g/L agar, рН 7.0–7.4) to assess fungal growth. The inoculated slants were incubated at 30°C for 24–72 h (LB) and at 24°C for 4–8 days (CD). The rest of the initial samples were stored at -80°C for molecular assays. The obtained microbial isolates were stored at 4°C and used for microscopy analysis, genomic DNA extraction, long-term storage collection, and inoculation of mock layers. For long-term storage, bacterial and fungal isolates were pre-grown in 5 ml of liquid media (LB or CD, respectively) in 50 ml tubes at 25°C for 24–72 h on a CERTOMAT®BS-1 shaker (Sartorius, Germany) at 230 rpm. After addition of glycerol (Merck, Germany) to a final concentration of 15%, cultures were aliquoted into 1.5 ml Eppendorf tubes, frozen in liquid nitrogen, and stored at -80°C.

### Characterization of microorganisms in initial samples, cultures, and infected mock layers

Microorganisms from initial samples grown on LB or CD agars or on mock layers were analyzed for texture, color of colonies, and hyphae morphology by macroscopic and microscopic observation as described previously with some modifications [18,19]. Light microscopy in native conditions was performed using a Carl Zeiss Jena microscope (Carl Zeiss, Germany) at ×1,000 magnification.

Identification of bacteria and fungi was performed by sequencing 16S rDNA or internal transcribed spacer (ITS) regions, respectively. Genomic DNA was isolated as described previously [20] with some modifications (Materials and methods 1.2 in S1 File). Bacterial 16S rDNA V3/V4 region was amplified using primers 341F (5'-CCTACGGGAGGCAGCAG-3') [21] and R806 (5'-GGACTACHVGGGTWTCTAA-3') [22]. Fungal ITS1 and ITS2 and 5.8S rDNA repeat units were amplified using primers ITS1 (5'-TCCGTAGGTGAACCTGCGG-3') and ITS4 (5'-TCCTCCGCTTATTGATATGC-3') [23]. PCR was performed in a Master Cycler amplifier (Eppendorf, Germany) using ScreenMix kit (Evrogen, Russia) or BioMaster LR HS-PCR kit (Biolab, Russia) at the following conditions: initial denaturation at 95°C for 3 min followed by 30 cycles of denaturation at 95°C for 30 s, primer annealing at 55°C (341F/R806) or 52°C (ITS1/ITS4) for 40 s, and elongation at 72°C for 30–50 s. PCR products were separated by electrophoresis in 1.5–1.8% agarose gels, purified using the CleanupMini kit (Evrogen), and sequenced using the Big Dye Terminator v.3.1 reagent kit (Applied Biosystems, Inc., USA) on a ABI PRIZM 3730 genetic analyzer (Applied Biosystems).

Culture-independent analysis was performed using next generation sequencing (NGS). The bacterial V3/V4 region was amplified with primers: 341F_BC (5'-TCGTCGGCAGCGTCAGATGTGTATAAGAGACAGCCTACGGGNGGCWGCAG-3') and R806_BC (5'-GTCTCGTGGGCTCGGAGATGTGTATAAGAGACAGGACTACHVGGGTATCTAATCC-3') and the fungal ITS2 region was amplified with primers: ITS86F_BC (5'-TCGTCGGCAGCGTCAGATGTGTATAAGAGACAGGTGAATCATCGAATCTTTGAA-3') and ITS4_BC (5'-GTCTCGTGGGCTCGGAGATGTGTATAAGAGACAGTCCTCCGCTTATTGATATGC-3'). Reactions were carried out with the Nextera XT Index kit (Illumina, Inc., USA) and quantified using the Qubit dsDNA HS Assay Kit (Invitrogen, Merelbeke, Belgium) in a Qubit Fluorometer prior to sequencing. Paired-end sequencing of the library was performed on an Illumina MiSeq sequencer (Illumina, Inc.) using the MiSeq Reagent Kit (v3) with the longest read length set to 2 × 250 base pairs (bp). The obtained sequences were compared against available sequences from GenBank (NCBI) databases using BLAST (https://blast.ncbi.nlm.nih.gov/Blast.cgi). The sequence data from Illumina MISeq for STG strains are publically available, as SRA records Accession: PRJNA606688, https://www.ncbi.nlm.nih.gov/sra/PRJNA606688.

### Preparation and inoculation of mock layers

Mock layers were developed with individually applied paints and varnishes used in tempera paintings of 16^th^ century and modern analogues, according to literature sources [7,8]; one of them was the passport of icon “The Church Militant” (S3 Fig). The list of materials used to develop mock layers and their numbers are provided in S1. 15 years old dry lime dock (48 x 60 cm) was covered with pavoloka (canvas). The ground layer (levkas) prepared by imposition the water solution of chalk and sturgeon glue, 70 g/l. On the smoothed-out levkas twenty different paint materials overplayed, 10 x 10 cm–each size, 2 cm–equal interval from each other (S4A Fig and S1 Table). Antiseptic pentachlorophenol sodium salt 1% (**SPCP**, Merck, USA), was added to layers 2–4 to protect against biodeterioration of sturgeon glue (or sturgeon glue with plasticizers). Thus obtained twenty mock layers numbered in the order of their application, from 1 to 20. For biodeterioration experiments the entire layout was mechanically fragmented onto 20 squares with single 10x10 cm applied material and levkas at the edges; then each square subdivided into 4 smaller pieces, containing about 5 x 5 cm of mock layer material and covered by ground layer from two sides (S4B–S4D Fig). After such preparation, the size of each subdivided piece allowed to use it for the infection by 4–5 cultivated microbial isolates by drop and dilution assay. Three of the four pieces could be applied for microbial growth while one of them remained untreated as a reference.

For microbial infection, fragmented pieces were placed into sterile Petri dishes and saturated with 0.3 ml H_2_O per 1 cm^3^ of piece at 25°C for 48 h. To avoid any contact with water of either side of the material, pieces with mock layers were placed on sterile hydrophobic spacers so that the lid of the Petri dish did not touch the surface of the mock layer. The isolates cultured on CD medium were pre-synchronized for the growth rate on agar using the drop and dilution method (spot analysis) described earlier [24]. The selected isolates were removed from oblique agar medium, resuspended in 0.9% NaCl to OD_600_ = 0.35, subjected to three consecutive 10-fold dilutions in 0.9% NaCl, and 1 μl of each dilution was inoculated into water-saturated mock layers, which were incubated for 4–12 days at 25°C and analyzed. Samples of each type (specific inoculum of a specific mock layer) were prepared in triplicate.

### Sample preparation for X-ray fluorescence (XRF) and micro-FTIR spectroscopy

On day 12 after inoculation, the applied materials with levkas and pavoloka were separated from the mock wooden board using a sterile scalpel and the central zone of inoculation (often showing visible signs of biodeterioration) with the surrounding areas were cut out. To analyze deep destruction of mock layers, the picked area was also washed in H_2_O with a cotton swab under sterile conditions until visible signs of microbial growth disappeared.

### XRF and data analysis

Intact and inoculated (before and after washing of the grown microbes) mock layers were analyzed by XRF spectroscopy using an ATRAX 800 spectrometer (Bruker, USA) under the following conditions: live time, 20 s; dead time, 4.1%; current, 602 mA; atmosphere, air; method, standard (Bayes); count rate, 20956 cps; voltage, 50 kV; optic, collimator 0.200. The data were processed and analyzed using the SPECTRA 7 Artax software (Bruker).

### Micro-FTIR spectroscopy and data analysis

Infrared spectra of intact and inoculated (before and after washing) mock layers were acquired using a LUMOS FTIR microscope (Bruker) in the wavenumber range from 600 to 4000 cm^-1^ in the attenuated total reflection mode; the probed area was 120 × 120 μm^2^. Each final spectrum represented an average of more than 500 individual spectra obtained from a given sample area. For each sample, the intact zone and the periphery and center of the inoculated zone were probed. A particular area to be probed was selected after obtaining a focused image of the sample surface. Spectra were processed and analyzed using the OMNIC software (Thermo Fisher Scientific, USA). To interpret the FTIR spectra of microorganisms, we used data obtained for pure cultures of *Bacillus amyloliquefaciens* [25], *Stenotrophomonas maltophilia* [26], or various species of genera *Aspergillus* [27,28], *Cladosporium* [29], *Ulocladium* [29], and *Bacillus* [30].

## Results and discussion

### Sampling

**Fig 1:**
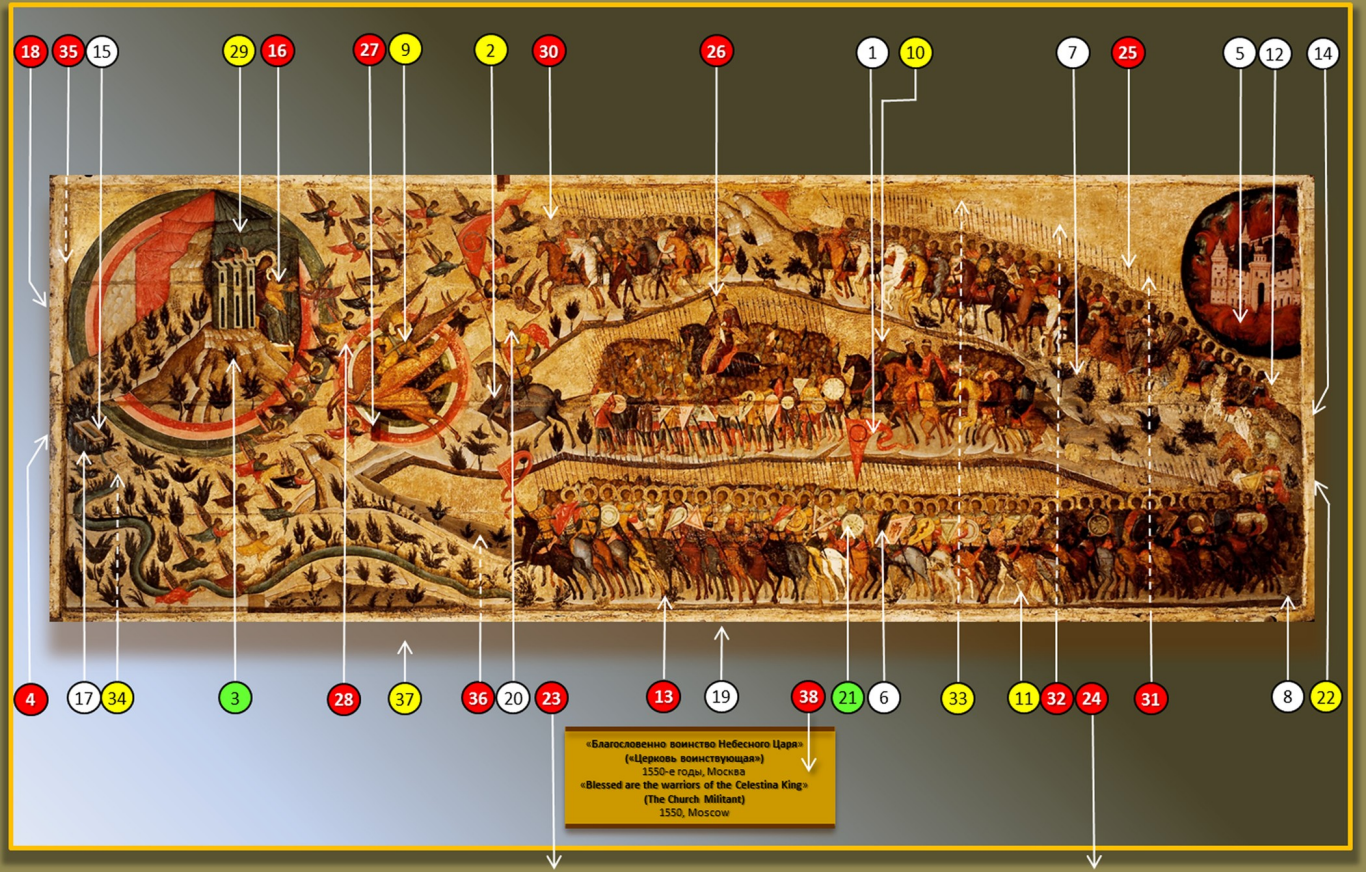
Sampling from icon “The Church Militant” and its surroundings. Arrows with solid lines indicate sampling from the front (tempera) side and arrows with dotted lines–sampling from the rear side. Sample numbers (1–38) are given in circles. Colors indicate growth on different culture media: yellow, growth only on LB, green–only on CD, red–on both LB and CD, and white–no growth on any.

The sampling process followed the requirements of conservation to produce the least possible impact on the structure and aesthetical value of the painting. Samples were obtained using non-invasive and micro-invasive methods; the minimum amount of sample required for different assays and sufficient to ensure representative analysis was collected. Samples from tempera paintings were obtained from different colored areas, cracks, and other zones with suspicious alterations. The detailed sampling maps for objects I–III are presented in Fig 1 and S5 Fig. Three exhibits of tempera painting were selected for analysis: two icons, “The Church Militant” and “Great Martyr St. Demetrius of Thessalonica”, and the limestone bust of “Saint George the Victorious” covered in tempera. The three objects were located in the risk zone, nearby the frontal wall (Hall №61, STG) expected to have internal biodeterioration according to STG visiting activity, which was monitored, in particular, by the maximum permissible levels of microorganisms (LLC "Mikosfera", Moscow, Russia). Fortunately, at the time of sampling (February 19, 2018), none of the analyzed pieces of art showed visible traces of microbial damage. However, after removal of the objects from Hall № 61 and dismantling fixing shields from the frontal wall, numerous and diverse signs of biodeterioration could be seen (S6 Fig). Such a critical state of Hall № 61 due to biodeterioration required major repairs and it was closed for reconstruction. Under such conditions, samples were taken on February 27, 2018 from sections of the frontal wall after dismantling panels, ceiling, cornices, and baseboards in Hall №61 and also in nearby Halls № 56 and 57, which are still open for visitors (S6 Fig).

In total, 106 samples were obtained, including 51 “tempera samples” (from objects I–III) and 55 “hall samples” (from the surrounding areas). Among the “tempera samples”, 23 were collected from object I (tempera side), 14 –from object II, and 14 –from object III; among “hall samples”, 15 were collected from object I (rear side and around), 30 –from Hall № 61 after conservation (29 –from the frontal wall and around), 5 –from Hall № 56, and 4 –from Hall № 57. The purpose of such sample collection was to determine if the infection clearly visible on the hall surfaces also spread to objects I–III (for example, as traces of fungal spores) and how dangerous it was in terms of biodeterioration risk for tempera painting. To address the first question, we performed comparative analysis of the microbiome in the collected samples by culture-dependent and culture-independent techniques. To answer the second question, we used different mock layers with materials used in tempera painting, inoculated them with cultures of microorganisms isolated from the collected samples, and analyzed by FTIR spectroscopy.

### Cultivation

Inoculation of LB and CD media with the initial samples produced a number of cultivable isolates (S7 Fig); as expected, fewer isolates were obtained from “tempera samples” than from “hall samples” (S8 Fig). Among “tempera samples”, slightly less microbial growth was observed for tempera-covered bust (object II, S8 2 Fig). “Hall samples” demonstrated higher germination rates in CD medium preferred by filamentous fungi and bacilli; however, isolates from the surroundings of object I also showed germination on LB. More than 90% of isolates from samples collected in the inner part of the frontal wall showed especially active growth on CD medium (S8 5 Fig). These data indicate that the analyzed halls present an unfavorable environment for exposition of tempera artifacts.

### Identification of microorganisms in samples and corresponding cultures

Sample-contaminating microorganisms revealed by culture-independent and culture dependent methods; the dominant isolated fungus affiliated with species of different genera within phyla Ascomycota (Table 1). The major cultivable fungi belonged to genera *Aspergillus*, *Cladosporium*, and *Ulocladium* and non-cultivable–to class *Saccharomycetes*. Bacterial isolates showed less difference depending on their culturability; thus, cultivable species predominantly belonged to phyla Firmicutes (*Bacillus*), Actinobacteria (*Microbacterium*, *Brachybacterium*), or Proteobacteria (*Stenotrophomonas*, *Achromobacter*) and non-cultivable–to phyla Proteobacteria (*Pelomonas*, *Pantoea*, *Stenotrophomonas)* or Firmicutes (*Clostridium*). Some microorganisms were defined as “uncultured” without species affiliation and were not included in this list. In some “hall samples”, *Ulocladium chartarum* was revealed. *Ulocladium* species are often found in water-damaged materials and their presence is an indicator of potentially wet environments or water-caused deterioration, which was observed inside the frontal wall in Hall № 61. Although *U*. *chartarum* was not detected in “tempera samples”, it was found in sample STG-36 from the rear side of object I (Fig 1); it was probably spread from the adjacent wet wall and persisted in the form of spores, which could be revived for growth by temporary changes in storage conditions and increased humidity. In contrast to *Ulocladium* preferring moist environments, the other two revealed genera, *Cladosporium* and *Aspergillus*, are able to tolerate drought, and some of their representatives can survive in extremely dry conditions [13,31,32]. Most of these species were found both in “tempera samples” and “hall samples” and, considering their physiological preferences, it is unclear whether the origin of contamination was the biodeteriorated wall or museum background infection.

**Table 1:** Phylogenetic analysis of ITS1, 5.8S ribosomal RNA and ITS2 sequences from STG samples.

Sample	Object	Phylum	Closest related type strain	Identity, %	GenBank accession number, submitted for current article
23B	I.2	*Ascomycota*	*Cladosporium parahalotolerans* culture CPC:22373 [MF473154.1]; [MF473153.1]; [MF473151.1]	100	MK268341.1
25G	I.1	*Ascomycota*	*Aspergillus versicolor* strain NHRC-FE077 [AJ937754.1]	100	MK260015.1
25W	I.1	*Ascomycota*	*Aspergillus creber* strain UTHSC 04–799 [LN898691.1]; [LN898689.1]	99	MK263226.1
26	I.1	*Ascomycota*	*Aspergillus sp*. AQGS [KP721563.1]	96	MK268343.1
S29	I.1	*Ascomycota*	*Aspergillus niger* isolate NPDF190-C1-F18 [MH024084.1]	100	MK271273.1
30	I.1	*Zygomycota*	*Mucor circinelloides* IFM 60050 [LC413613.1]	100	MK260195.1
52B	II	*Ascomycota*	*Cladosporium halotolerans* strain HNC20-79 [KX867546.1]; [KT989417.1]	100	MK258720.1
57	III	*Ascomycota*	Aspergillus creber strain UTHSC 10–582 [LN898694.1]	100	MK266993.1
86	IV	*Ascomycota*	*Aspergillus versicolor* strain NHRC-FE078 [AJ937755.1]	100	MK262781.1
91	IV	*Ascomycota*	*Cladosporium halotolerans* strain Y8_1 small [MH063019.1]	100	MK265717.1
92	IV	*Ascomycota*	*Cladosporium halotolerans* strain UTHSC DI-13-249 [LN834373.1]	99	MK264775.1
93B	IV	*Ascomycota*	*Cladosporium parahalotolerans* culture personal: Jos: Houbraken: DTO: 324-B7 [MF473169.1]; [MF473168.1]; [MF473166.1]; [MF473165.1]	99	MK262909.1
96	IV	*Ascomycota*	*Simplicillium lamellicola* isolate L12C [FJ713056.1]	100	MK262921.1
103	IV	*Ascomycota*	*Cladosporium cladosporioides* isolate A1S1-1 [KJ767065.1]	100	MK262923.1
106	IV	*Ascomycota*	*Aspergillus amoenus* strain UTHSC 09–2582 [LN898677.1]	100	MK268342.1

Since “tempera samples” were collected from different color spots and problematic areas (including crack zones), it was important to characterize microorganisms present in the initial probes and corresponding cultures. Thus, two phylogenically very close *Aspergillus* species, *A*. *versicolor* and *A*. *creber* [33] (GenBank accession nos. MK260015.1 and MK263226.1, respectively), which were found in the crack of icon “The Church Militant”, are of concern (sample 25, Fig 1). A noticeable feature revealed in the sample from the black tempera zone of object I was the presence of black *A*. *niger* (GenBank accession no. MK271273.1; sample 29, Fig 1), which, because of the color camouflage, was hidden from the restoration service for some time. It is known that certain types of filamentous fungi, in particular *Aspergillus*, are capable to produce hundreds of different secondary metabolites [34]. Many of them are colored and negatively affect paintings [35], but can be quickly detected by professional staff. However, if the color of the fungal pigment is similar to that of the affected area, visual detection is complicated, leading to deeper infection. Zygomycota *Mucor circinelloides* (GenBank accession no. MK260195.1) isolated from the “tempera side” of object I (sample 30, Fig 1) is also of concern because of its possible rapid growth if conditions turn favorable. However, representatives of this phylum were not detected in the other samples, suggesting that the *Mucor* infection was not a result of contamination from the walls.

It should be noted that one of the frequent bacterial isolates was *Stenotrophomonas* sp. detected in “tempera samples” together with *Aspergillus* (probes 25, 27), suggesting a possible interaction between the two microorganisms, as previously shown for *S*. *maltophilia* and *A*. *fumigatus* [36]. A wide spectrum of enzymatic activity demonstrated by various *Stenotrophomonas* spp. enables them to metabolize substrates of diverse chemical nature [37], which may provide survival benefits for xerophilic *Aspergillus* such as *A*. *creber* or *A*. *versicolor* [13].

The predominant prokaryotes associated with the deterioration of internal sections of the frontal wall and adjacent ceilings were *Bacillus* spp., including their major representative *B*. *amyloliquefaciens*, which also caused characteristic redness of the wall surface. In a number of CD cultures, *B*. *amyloliquefaciens* coexisted with *Aspergillus*; thus, in cultured sample 86, *B*. *amyloliquefaciens* was present together with *A*. *versicolor*. The high growth rate of “frontal wall” isolates on CD agar was due to *Bacillus*, which could effectively proliferate on this medium (S8 *5* Fig). *Bacillus* spp., in particular *B*. *amyloliquefaciens*, were also found among “tempera samples”, which may indicate the spread of biodegradation from the wall to tempera exhibits.

### Analysis of intact mock layers

To assess potential destruction of tempera paintings by the microbiome existing in STG, we employed artificial models, so-called mock layers, which were developed in the restoration workshop of STG using a variety of materials applied in tempera painting of 16^th^ century as well as their modern analogues. Prior to inoculation of mock layers, cultivable isolates were pre-synchronized for the growth rate on CD agar to ensure simultaneous cultivation on a single piece of mock layer (four pieces were used for each layer). One sample (number 30) containing *M*. *circinelloides* was excluded from the experiment because this fungus demonstrated rapid growth, covering the entire CD-agar dish with aerial mycelium in 3–4 days and interfering with the growth of other samples. As a result, two groups (sets) of cultivated samples, representing dominant microorganisms isolated from STG, were obtained for simultaneous inoculation of mock layers (S9 Fig and S2 Table). One set with five inoculums (probes 103, 106, 36, 93B and 93W; positions 1.1–1.5, respectively) and the other set with four inoculums (probes 25G, 86, 57 and 96; positions 2.1–2.4, respectively) used for infection of two same fragments from each mock layer.

**Fig 2:**
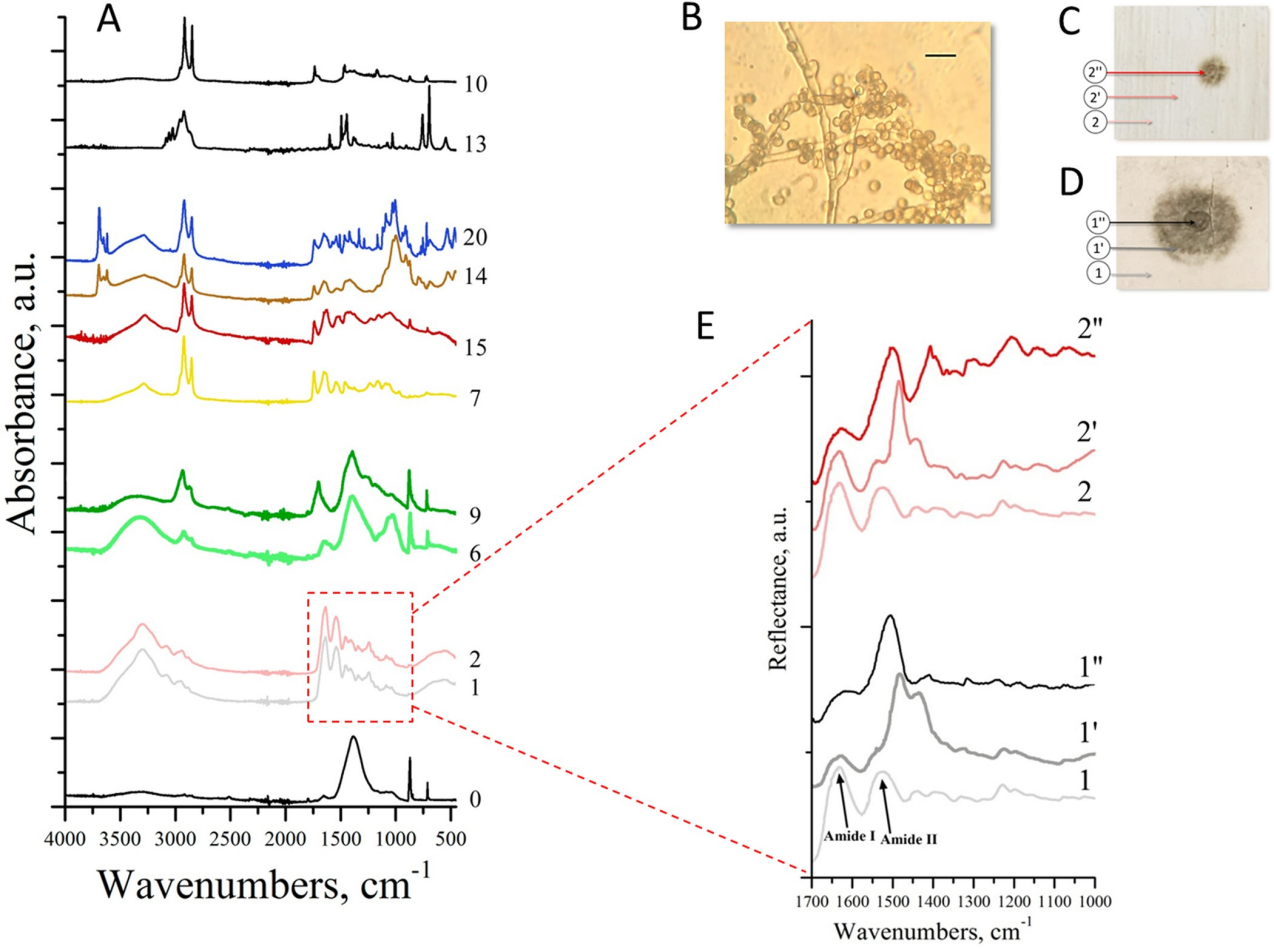
Characterization of mock layer models using micro-FTIR spectroscopy. (A) FTIR absorption spectra for intact mock layers. (B)–(E) Modeling of sturgeon glue biodeterioration with cultured isolate 93B (containing *C*. *parahalotolerans*). Light microscopy analysis of inoculum 93B; scale bar = 10 μm (B). Spectral properties of sampling zones: 1, 2 –intact zones; 1', 2'–periphery of the inoculated zone (according to the growth on mock layer 1); 1'', 2''–center of the inoculated zone (C–E). Visible biodeterioration of mock layers 1, 2 with inoculum 93B after 12 days at 25°C (C, D). FTIR spectra revealing biodeterioration (E). Levkas, 0; glues: 1, 2 –sturgeon glue without and with 1% SPCP, respectively; plasticizers: 6 –gum arabic, 9 –rosin; tempera paints: 7 –egg emulsion, 14 –egg tempera with ochre, 15 –egg tempera with cinnabar, 20 –egg manufacturing tempera “Rowney” (Monestial Blue Phthalo); varnishes: 10 –wax-oil mastic, 13 –Dammar varnish (see also S1 Table). Colors of spectra for tempera mock layers (7, 14, 15, and 20) correspond to those of the layers.

We also performed preliminary evaluation of mock layers by elemental and spectral analyses using XRF (S10 Fig and S3 Table) and micro-FTIR (Fig 2A) spectroscopy, respectively. XRF spectroscopy was a powerful tool for characterization of mock layer structures (Results and discussion 2.1 in S2 File). In case of levkas, we used the remaining open areas between applied materials (Materials and methods 1.3 in S1 File). XRF revealed that Ca was the major detectable element in levkas, which also had trace amounts of Fe; these results are consistent with the method of levkas manufacturing from chalk as the main material (Materials and methods 1.3 in S1 File). Elemental analysis also showed that egg tempera from egg yolk, when applied on levkas (S4A Fig, mock layer 7), did not produce any changes in sample composition (S3 Table). For tempera with pigments ochre, cinnabar, or copper phthalocyanine (CuPc), which produced yellow-brownish, red, and blue shades in mock layers 14, 15, and 20, respectively, component analysis revealed the appearance of peaks typical for iron, mercury, and copper, respectively, on the Ca background from levkas (S10 Fig).

FTIR spectra could be divided into five types: four for mock layers and one for levkas (Fig 2A). The spectrum of levkas differed from those of mock layers by showing a predominant absorption peak characteristic for Ca. However, FTIR spectra for plasticizers (mock layers 6, 9) had a number of absorption peaks very close to those for levkas, probably because these materials were semi-transparent to FTIR spectroscopy and the resultant spectrum presented a superposition of plasticizer and levkas spectra. For sturgeon glue mock layers, the addition of antiseptic (1% SPCP) did not affect their spectral properties; one of the most important spectral features of these samples (mock layers 1, 2) was the presence of absorption bands characteristic for amides I and II. Spectra of tempera paints (mock layers 7, 14, 15, 20) were very similar; however, it was possible to subdivide them into two groups according to differences in the 750–1250 cm^-1^ zone: mock layers 7, 15 and 14, 20. Mock layers 7, 14, 15 were manufactured from materials used in 16^th^ century, whereas mock layer 20 was “Rowney” (Monestial Blue Phthalo)–egg-containing tempera with CuPc first manufactured in 1927; its FTIR spectrum was very close to those of other tempera paints with a distinct CuPc profile [38].

### Infection of mock layers

**Fig 3:**
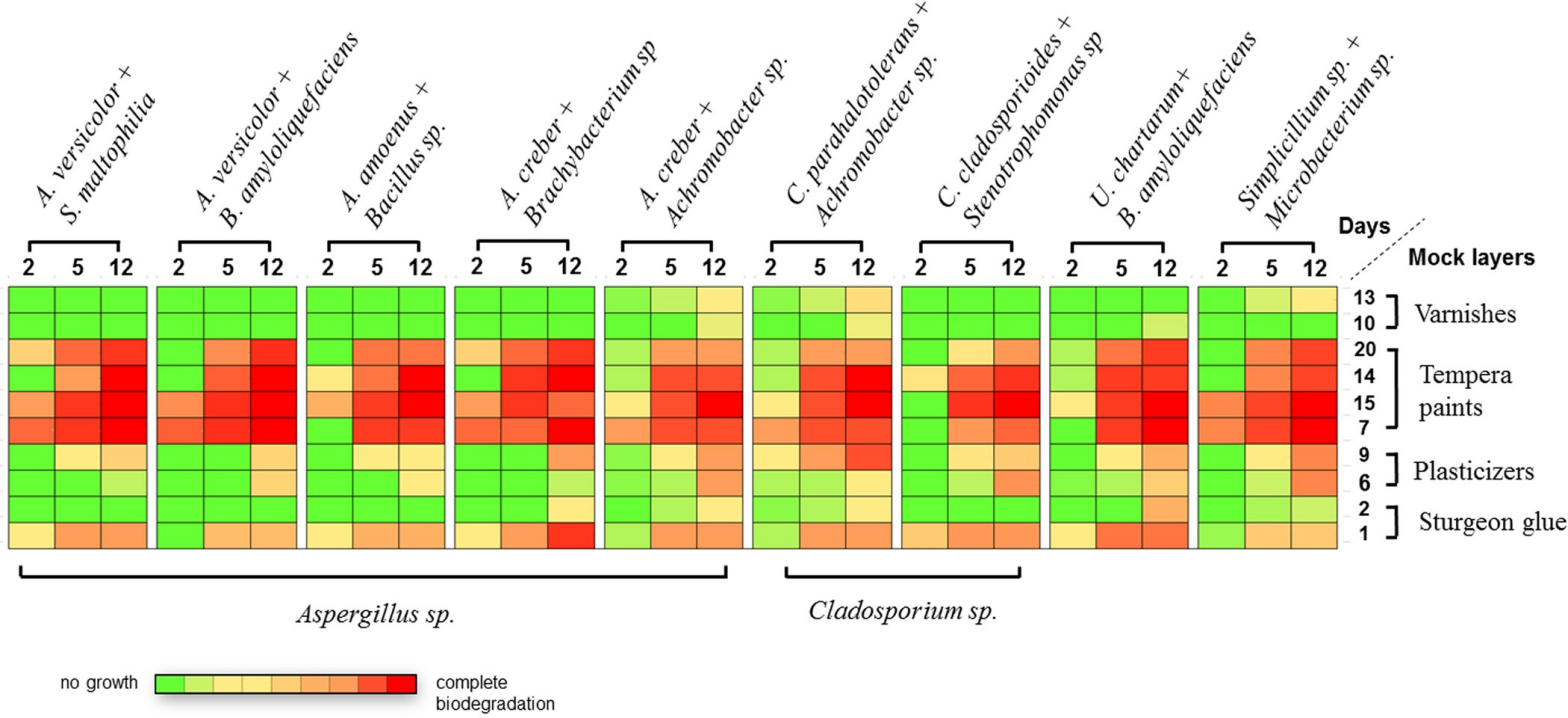
Culture growth on mock layers and biodeterioration dynamics. Mock layers (indicated on the right) were inoculated with microorganisms (indicated on the top), incubated for 2, 5, or 12 days at 25°C, and analyzed for microbial growth.

**Fig 4:**
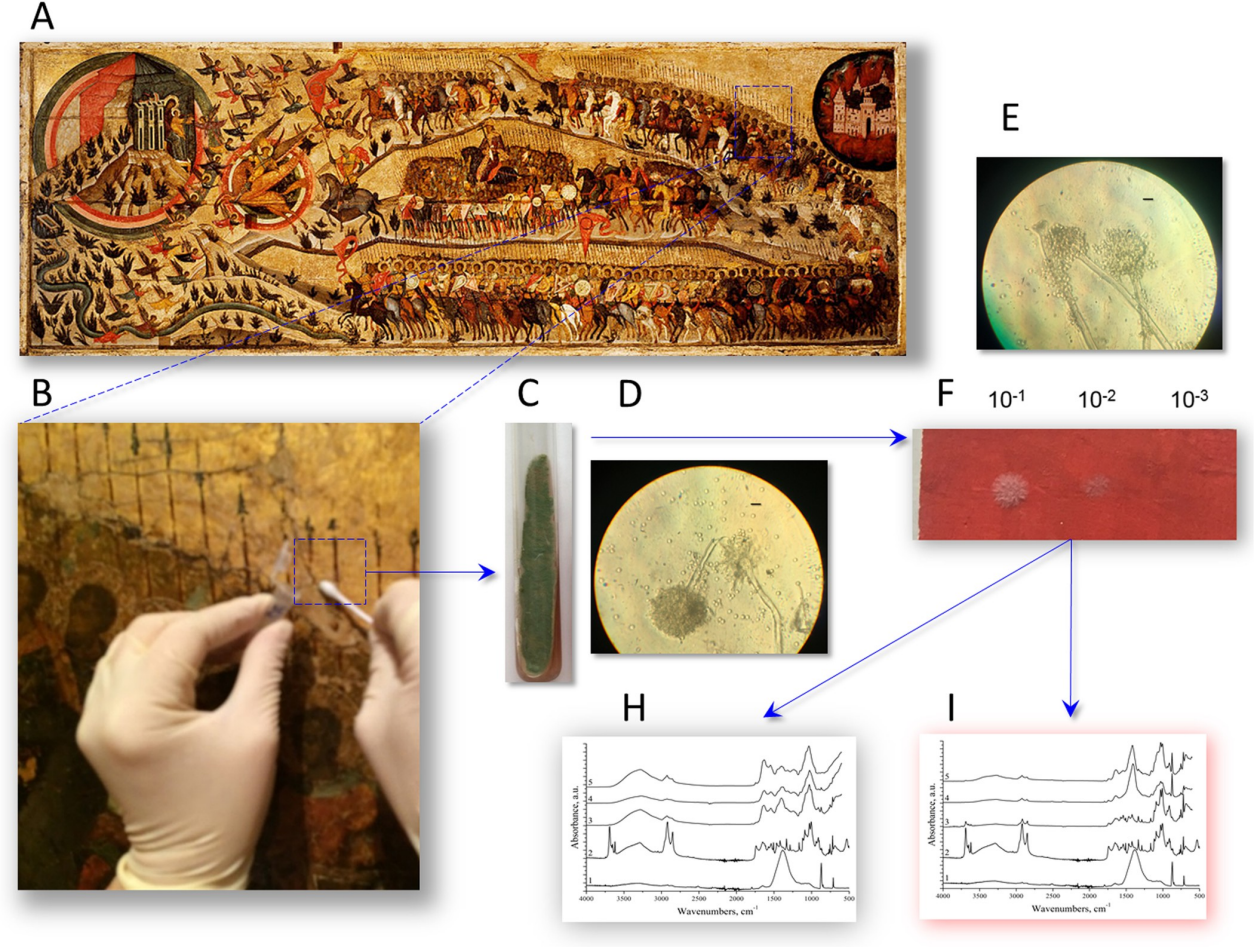
Analysis of biodeterioration in tempera paintings. (A) Icon “The Church Militant” (object I). (B) Collection of sample 25 from the crack of object I. (C) Sample cultivation on slant CD agar. (D and E) Light microscopy images of sample 25 growth on CD agar (D) and on the surface of mock layer 15 (E); scale bar = 10 μm. (F) Drop and dilution assay for inoculum 25G growth on mock layer 15 (48 h at 25°C). (H and I) Micro-FTIR spectroscopy analysis of biodeterioration in mock layer 15 before (H) and after (I) removal of the 25G-produced microbial sheet. FTIR spectra: 1 –intact levkas, 2 –intact mock layer 15, 3 –periphery of the inoculated zone; 4 –areola zone; 5 –center of the inoculated zone.

Each of 10 selected mock layers was inoculated with 9 selected cultured samples containing mixtures of isolates grown on CD medium by the drop and dilution method (S2 Table). The inoculums grew on pre-saturated mock layers in sterile Petri dishes, 25°C. Direct examination confirmed the ability of the inoculated fungi to colonize mock layers, as they produced mycelia and conidia; for some samples, visible growth was observed already after 36–48 h (S11 Fig). At day 12 post inoculation, the colonies were analyzed for macroscopic and microscopic features (Fig 4E). Dynamics of biodegradation (days 2, 5, and 12) indicated that the most vulnerable part of tempera multi-layered samples was tempera paint, its most valuable component (Fig 3). Among tempera paints, natural egg tempera without or with cinnabar pigment (mock layers 7 or 15, respectively) were the best substrates for microbial growth as they were quickly (in 2 days) colonized with 8 of 9 samples. Perhaps, the presence of Fe (ochre pigment) or CuPc in the other tempera mock layers inhibited fungal growth at the early stage; although Fe and Cu are essential trace elements required for microbial metabolism, they are toxic at increased concentrations [39,40]. On the other hand, CuPc is stable and not biodegradable [41], suggesting that low levels of CuPc-released Cu may not be sufficient to inhibit microbial growth when added to egg emulsion (S11A and S11D Fig). However, by day 5 post inoculation the inhibitory effects of all pigments were overcome, and by day 12, all tempera mock layers were completely bio-damaged (Fig 3). The same effect was observed on day 12 for mock layer 1 manufactured with sturgeon glue; the addition of 1% SPCP preserved the layer from biodeterioration by 4 of 9 inoculates. The results among plasticizers were similar, although the biodeterioration of rosin (mock layer 9) was slightly stronger than that of gum arabic (mock layer 6) on day 12. The best antimicrobial resistance was demonstrated by varnishes, which is consistent with the main purpose of using varnishes as protectors of the inner layers of tempera paintings from damage. According to our data, the selected varnishes could effectively protect against biodeterioration, although with some exclusions (Fig 3): Dammar varnish (mock layer 13) inoculated with samples 93B, 93W, or 96 and wax-oil mastic (mock layer 10) inoculated with samples 36, 93B, or 93W leaked on day 12.

To illustrate the risks of bio-damage in tempera painting, we reconstructed the biodeterioration process by analyzing the microbial growth on individual paints and varnishes (mock layers) (Fig 3). For the convenience of interpretation, the examined mock layers are arranged in the order of their application when a tempera painting is created. Our experiments demonstrated that tempera paints, the most valuable inner layers of painting, were at the same time the most vulnerable and presented a good nutrient substratum for growth of the isolated microorganisms. We analyzed tempera paints with red, brownish-yellow, and blue pigments (Fig 3, mock layers 15, 14 and 20, respectively), which are fundamentally important for the formation of color palette; they are created by the addition of metals or organic pigments, which obviously do not protect tempera paints against biodegradation. Sturgeon glue with antiseptic SPCP (Fig 3, mock layer 2) and especially varnishes (Fig 3, mock layers 13, 10) used as top and bottom layers, respectively, showed stronger resistance to microbial colonization and, thus, can preserve paints from biodegradation. However, microorganisms can penetrate these protective layers through cracks due to mechanical stress and get access to the inner tempera paint layer. Therefore, the presence of *A*. *versicolor*, *A*. *creber*, and *S*. *maltophilia* in sample 25 in the crack of icon “The Church Militant” causes serious concern (Fig 4).

Overall, the 9 inoculates demonstrated similar patterns of bio-damage, despite different microbial composition (S2 Table). The main differences were observed for mock layers created with varnishes, as only 3 out of the 9 cultures could grow on the varnish-covered surface; among them, cultures 93W and 93B grew both on Dammar varnish (mock layer 13) and wax-oil mastic (mock layer 10). Since no other *Aspergillus* (including *A*. *creber* from sample 57) or *Cladosporium* demonstrated the ability to grow on varnishes, the enzymatic activity in degrading mock layer components in samples 93W and 93B could be attributed to *Achromobacter* sp. common for both samples. Previously, it was demonstrated that mock paintings fully imitating oil painting on canvas were biodeteriorated by *Arthrobacter* sp. and *Penicillium* sp. in combination but not by each species alone [1]. In this study López-Miras et al. (2013a) used fully mocked models of painting rather than separate mock layers, with natural resin shellac as varnish covering two types of mineral oils with pigments and other layers; however, such a model does not allow determination of the material degraded through joint enzymatic activity; possibly, it was varnish. In our study, CD-cultured inoculums represented mixtures of fungi and bacteria; among them, samples 36 and 96 demonstrated growth on varnishes: wax-oil mastic (mock layer 10) and Dammar varnish (mock layer 13), respectively. The addition of 1% SPCP increased the resistance of sturgeon glue against all 9 tested microbial cultures (Fig 3); still, initial stages of biodeterioration were detected for 5 out of 9 samples on day 12, which is higher than the rate observed for varnishes. Therefore, it is necessary to develop new more effective but at the same time safe antiseptics.

In this study, we demonstrated, for the first time, that *S*. *maltophilia* could grow in combination with xerophilic *Aspergillus* fungi *A*. *creber* or *A*. *versicolor*. *S*. *maltophilia* isolates could utilize a wide variety of substrates [37], which is likely due to a broad spectrum of enzymatic activities detected in *Stenotrophomonas* spp., enabling the bacteria to metabolize different materials. *S*. *maltophilia* is pathogenic for humans, and in many clinical cases co-infection with *S*. *maltophilia* and *A*. *fumigatus* was observed [36]. Iron is required for *S*. *maltophilia* biofilm formation and virulence [26]; however, after 48 h inoculum 25G containing *Stenotrophomonas* and *Aspergillus* grew better on tempera paints with cinnabar, CuPc, or without any pigments than on those with Fe-containing ochre, which was due to dominant fungal growth (S11.*6* Fig). *U*. *chartarum* is well known to be involved in biodeterioration of organic and inorganic substrates; it can invade surfaces containing very little substrate, thus posing a risk of degradation for various materials, including cultural artifacts [42,43]. In our study, *U*. *chartarum* demonstrated growth inside CD agar and mock layers (S9 and S11 Figs), suggesting its possible role in biodeterioration of tempera painting.

### Micro-FTIR analysis of biodeterioration

**Fig 5:**
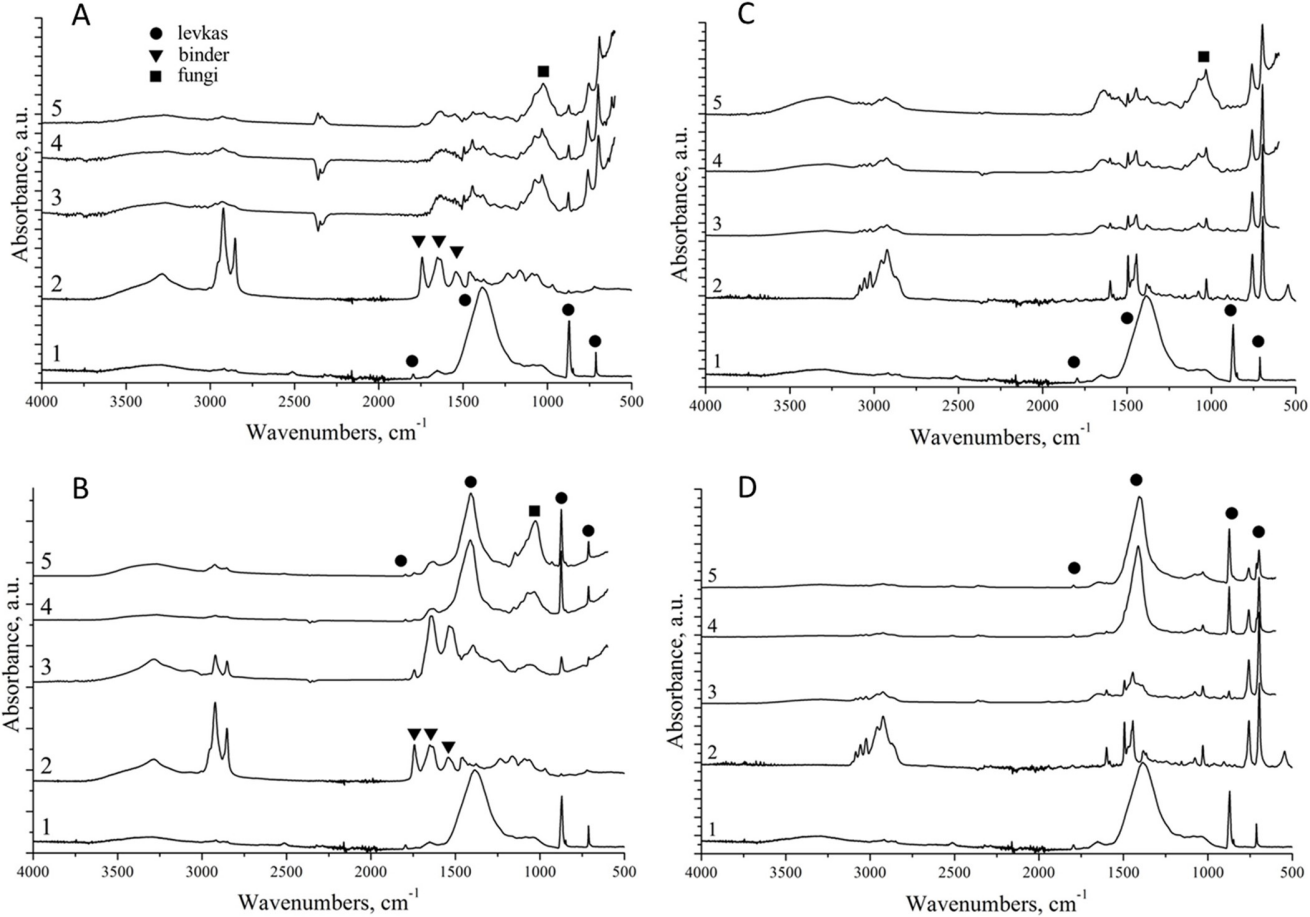
Micro-FTIR spectroscopy of inoculated mock layers. Analysis was performed for inoculated samples incubated for 12 days at 25°C. (A and B) Mock layer 7 inoculated with sample 106 containing fungus *A*. *amoenus*. (C and D) Mock layer 13 inoculated with sample 93W containing fungus *A*. *creber*. (A and C) Spectra with microbial sheets. (B and D) Spectra after removal of microbial sheets. 1 –intact levkas, 2 –intact mock layer 15, 3 –periphery of the inoculated zone; 4 –areola zone; 5 –center of the inoculated zone. Absorption peaks: circles–levkas (ground layer), triangles–binder (egg emulsion), squares–fungi.

The principle scheme of preparing samples for micro-FTIR analysis is shown for sample 25G: probe isolation from a crack in object I, culture on CD agar, and infection of mock layer 15 (Fig 4). For visible spots of biodeterioration, micro-FTIR analysis was performed in mock layers with microorganisms and after their removal. In case of active microbial growth on the surface, the resulting microbial sheet was not transparent for FTIR spectroscopy and we could obtain only spectra characteristic for the inoculated microorganisms. Thus, *A*. *amoenus* (inoculum 106) or *A*. *creber* (inoculum 93W) produced a spectrum profile typical for *Aspergillus*, with a double band near 1000 cm^-1^ (squares, Fig 5) corresponding to the maximum absorption of *Aspergillus* polysaccharides [27,28]. After removal of the microbial sheet, the measured FTIR spectra indicated the level of biodeterioration. For both initial and washed samples, FTIR spectroscopy was performed in three zones: the center of inoculation (corresponding to the area of maximum growth), the areola zone (between colony center and edge), and the periphery, which enabled spatial analysis of microbial growth and biodegradation spread.

Fig 5 shows FTIR spectra of levkas and two mock layers inoculated with *Aspergillus* fungi; the data for other inoculated models are shown in S12–S15 Figs. *Aspergillus* was present on the surface of both mock layers (7 –egg emulsion and 13 –Dammar varnish), as was evident by the masking of bands belonging to the egg emulsion layer (Fig 5A *2*–*5*) and appearance of the *Aspergillus* double band in the 900–1200 cm^-1^ range (Fig 5A and 5C). The presence of some characteristic peaks in the 1400–1500 cm^-1^ range for Dammar varnish indicated that fungal growth on this material was insufficient for complete coverage of its characteristic FTIR bands, even at the center of inoculation (Fig 5C *2*–*5*). After removal of the microbial sheet from the egg emulsion mock layer, peripheral degradation of the material was detected, because absorption peaks for carbonyl groups (1700–1750 cm^-1^) were significantly decreased, whereas the intensity for amide I and amide II peaks was unchanged (Fig 5B *2*, *3*). However, the characteristic *Aspergillus* double band (900–1200 cm^-1^) was not detected, which may indicate the initial stage of biodegradation, when the fungal mycelium did not yet penetrate deeply into levkas. In contrast, almost complete degradation of the tempera material in areola and inoculation zones was revealed after microbial sheet washing, as evidenced by almost complete disappearance of carbonyl, amide I, and amide II bands. Removal of the microbial sheet and bio-degradation of the egg tempera layer uncovered the next layer, i.e., levkas, for FTIR spectroscopy (Fig 5B *4*, *5*), which revealed four peaks characteristic for calcium carbonate (levkas component) in the 600–1800 cm^-1^ range. Along with peaks from levkas and traces of the remaining tempera, typical absorption bands of *Aspergillus* were clearly observed; given that the top microbial sheet was washed out, these spectra strongly suggest penetration of fungi into the depth of the ground layer, levkas. The intensity of the double peaks in the 900–1200 cm^-1^ range showed that fungal growth was higher in the inoculation zone than in the areola zone. These results indicated that our methodical approach allows spectral detection of different stages in egg tempera biodeterioration.

FTIR spectral analysis of the washed mock layer 13 with Dammar varnish revealed slight degradation on the periphery and full degradation in the areola and central zones (Fig 5D *3–5*). However, microorganisms in the washed samples were not detected, due to the absence in spectra the additional (to Dammar varnish or levkas) bands, indicating that microbes did not penetrate into the ground layer. These results suggest that Dammar varnish, along with rosin, is a good barrier preventing fungal spread (Figs 5C and 5D and S13C and S13D).

FTIR spectral analysis of biodeterioration of sturgeon glue (mock layers 1, 2) by inoculum 93B (containing fungus *C*. *parahalotolerans*) revealed that the addition of 1% SPCP inhibited microbial growth even in the inoculation zone, as evident by increased intensity of the amid I band in the sample with antiseptic (Fig 2E *1''*, *2''*), which corresponds to sturgeon glue (Fig 2E *1*, *2*). The signal for the amid II band was completely masked by *Cladosporium* signals [29] in the center of the inoculated zone of both layers 1 and 2, but not in the periphery of mock layer 2 (Fig 2E), and the visible area of microbial growth on the mock layer with antiseptic was much smaller (Fig 2C and 2D). Thus, the site in mock layer 2 (Fig 2C *2'*) which corresponded (by the distance from the inoculation center) to the peripheral zone of microbial growth in mock layer 1 (Fig 2D *1'*), did not show any traces of biodeterioration. However, the FTIR spectrum for this zone clearly revealed a partially disappearing amid II band (from sturgeon glue) and the appearance of additional peaks (Fig 2E *2'*), which corresponded to growing infection as evident from comparison with the FTIR spectrum for the inoculation zone with clear microbial growth (Fig 2E *2''*). These results clearly demonstrate the power of FTIR spectroscopy to detect even invisible traces of biodeterioration.

## Conclusions

In this study, we performed analysis of biodeterioration processes in tempera paintings using a comprehensive set of materials, from sturgeon glue to plasticizers, tempera paints, and varnishes, which allowed recapitulation of biodegradation processes in the art museum environment and analysis of their dynamics.

## Supporting information

S1 Fig Sampling in Hall 61, view to the frontal wall (16^th^ century icon painting, STG, Moscow, Russia). Numbers in ovals or circles correspond to sample numbers. Samples: 1–22, 25–30 –object I, “tempera side”; 23, 24, 36–38 –object I, nearby; 31–35 –object I, rear side; 42–55 –object II; 56–69 –object III; 70–88 –frontal wall; 89–95 –ceiling and cornices; 96–98 –left wall; 99 –dismantled front wall panel.
(TIF)

S2 Fig Hall 61, sampling from rear wall after closing for exhibition due to biodegradation. Arrow indicate site for sample; number in circle corresponds to the sample number (100).
(TIF)

S3 Fig The passport of the icon “The Church Militant”. (A) Scan from the original document from the archive of STG (text–in Russian). (B) English translation of the original passport.
(TIF)

S4 Fig Development and defragmentation of mock layers. (A) Original desk with applied mock layers. (B-D) Defragmented mock layers: (B) mock layer 4; (C) mock layer 16; (D) mock layer 18.
(TIF)

S5 Fig Sampling from object II, III. Samples distribution with: (A) bust of the “Saint George the Victorious”, (B) icon “Great Martyr St. Demetrius of Thessalonica”. Light microscopy analysis after sampling from objects I-III, scale bar = 10 μm (the species identity was determined after genotyping): (C) *Aspergillus creber*, (D) *Mucor circinelloides*, (E) *Ulocladium chartarum*. Arrows with solid lines correspond for sampling from tempera (front) side; arrows with doted lines–for sampling from rear side. Numbers in circles correspond to sample numbers: 42–55 –object II; 56–69 –object III. Cultivation on LB and CD media: yellow circles–growing only on LB medium, green circles–growing only on CD, red circles–growing on LB and CD, white circles–no growth on both mediums.
(TIF)

S6 Fig Sampling in hall 61 (after dismantling the exhibits and dismantling the front wall) and in the surrounding areas. Arrows indicate sampling sites. Numbers in circles correspond to the sample numbers: 70–99 –Hall №61, frontal wall and nearby; 100 –Hall 61, rear wall; 101–104 –Hall 56; 105–109 –Hall 57. Cultivation on LB and CD media: yellow circles–growing only on LB medium, green circles–growing only on CD, red circles–growing on LB and CD, white circles–no growth on both media.
(TIF)

S7 Fig Cultivation on CD-agar medium STG-isolates, 25°C. (A, C, E and G)–initial isolates growth on slant agar. (B, D and F)–light microscopy analysis, scale bar = 10 μm. Samples and corresponding species after genotyping: (B and C)–sample 27 (*Aspergillus versicolor*); (D and E)–sample 96 (*Simplicillium lamellicola*); (F and G)–sample 103 (*Cladosporium cladosporioides*).
(TIF)

S8 Fig Cultivation on slant agar media after sampling. Samples growth from: 1 –object I, tempera side; 2 –object II; 3 –object III; 4 –object I, rear side and nearby; 5 –frontal wall (internal part) and ceilings. Solid line corresponds to maximum percent number of cultivable isolates, obtained from tempera painting objects, on LB medium; dotted line–to maximum number of cultivable isolates, obtained from tempera painting on CD medium.
(TIF)

S9 Fig Cultivation on CD-agar medium of STG-isolates. Drop and dilution assay, 25°C. (A and C) First set of isolates; (B and D) Second set of isolates. (A and B)– 3, (C)– 4, (D)– 12 days. Samples: 1–103; 2–106; 3–36; 4 – 93B; 5 – 93W; 6 – 25G; 7–86; 8–57; 9–96.
(TIF)

S10 Fig XRF analysis of mock layers. X-ray fluorescence spectra and probes for sampling (analyzing site is point into the center of red box). (A) 7 mock layer, (B) 14 mock layer, (C) 15 mock layer, (D) 20 mock layer.
(TIF)

S11 Fig Sample growth on the surface of mock layers. Drop and dilution assay, 48 h, 25°C. Mock layers: (A) 7 mock layer, (B) 14 mock layer, (C) 15 mock layer, (D) 20 mock layer. Samples: 1–103, 2–106, 3–36, 4 – 93B, 5 – 93W, 6 – 25G, 7–86, 8–57, 9–96.
(TIF)

S12 Fig Micro-FTIR spectroscopy analysis after inoculation of mock layers, 12 days, 25°C. (A and B) Mock layer 7, inoculum–sample 36 (with fungus *U*. *chartarum*); (C and D) Mock layer 7, inoculum–sample 93W (with fungus *A*. *creber*). (A and C) Initial probes with microbe topsheet; (B and D) After washing from microbes. FTIR spectra: 1 –intact levkas, 2 –intact mock layer 15, 3 –periphery of inoculated zone; 4 –areola zone; 5 –center of inoculated zone.
(TIF)

S13 Fig Micro-FTIR spectroscopy analysis after inoculation of mock layers, 12 days, 25°C. (A and B) Mock layer 9, inoculum–sample 93W (with fungus *A*. *creber*); (C and D) Mock layer 14, inoculum–sample 103 (with fungus *C*. *cladosporioides*). (A and C) Initial probes with microbe topsheet; (B and D) After washing from microbes. FTIR spectra: 1 –intact levkas, 2 –intact mock layer 15, 3 –periphery of inoculated zone; 4 –areola zone; 5 –center of inoculated zone.
(TIF)

S14 Fig Micro-FTIR spectroscopy analysis after inoculation of mock layers, 12 days, 25°C. (A and B) Mock layer 15, inoculum–sample 106 (with fungus *A*. *amoenus*); (C and D) Mock layer 15, inoculum–sample 93B (with fungus *C*. *parahalotolerans*). (A and C) Initial probes with microbe topsheet; (B and D) After washing from microbes. FTIR spectra: 1 –intact levkas, 2 –intact mock layer 15, 3 –periphery of inoculated zone; 4 –areola zone; 5 –center of inoculated zone.
(TIF)

S15 Fig Micro-FTIR spectroscopy analysis after inoculation of mock layers, 12 days, 25°C. (A and B) Mock layer 20, inoculum–sample 93W (with fungus *A*. *creber*); (C and D) Mock layer 20, inoculum–sample 25G (with fungus *A*. *versicolor*). (A and C) Initial probes with microbe topsheet; (B and D) After washing from microbes. FTIR spectra: 1 –intact levkas, 2 –intact mock layer 15, 3 –periphery of inoculated zone; 4 –areola zone; 5 –center of inoculated zone.
(TIF)

S1 Table Materials, used for application of mock layers. ^*^ Used materials applied over the levkas layer for mock layer preparation.
(DOCX)

S2 Table Cultured microorganisms used for inoculation of mock layers. (DOCX)

S3 Table Component composition of mock layers according XRF spectroscopy. (DOCX)

S1 File Materials and methods. (DOCX)

S2 File Results and discussion. (DOCX)
