# Supplementary figures and images for: Detection of potential biodeterioration risks for tempera painting in 16th century exhibits from State Tretyakov Gallery
Source: PLoS One. 2020 Apr 2;15(4):e0230591. doi: 10.1371/journal.pone.0230591 (PMC7117676; doi:10.1371/journal.pone.0230591)

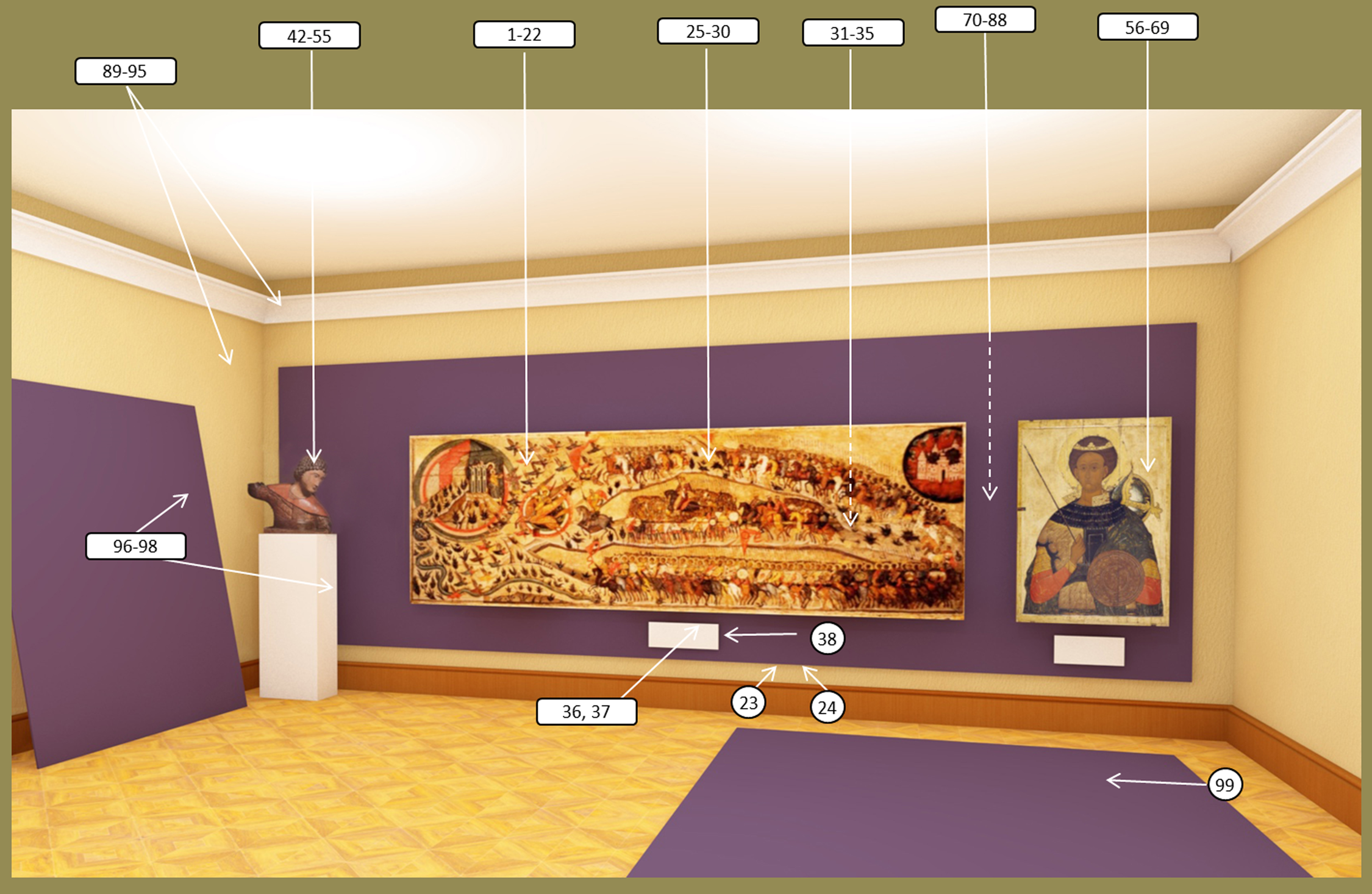

Supplement: S1 Fig — Numbers in ovals or circles correspond to sample numbers. Samples: 1–22, 25–30 –object I, “tempera side”; 23, 24, 36–38 –object I, nearby; 31–35 –object I, rear side; 42–55 –object II; 56–69 –object III; 70–88 –frontal wall; 89–95 –ceiling and cornices; 96–98 –left wall; 99 –dismantled front wall panel. (TIF) [file pone.0230591.s001.tif]

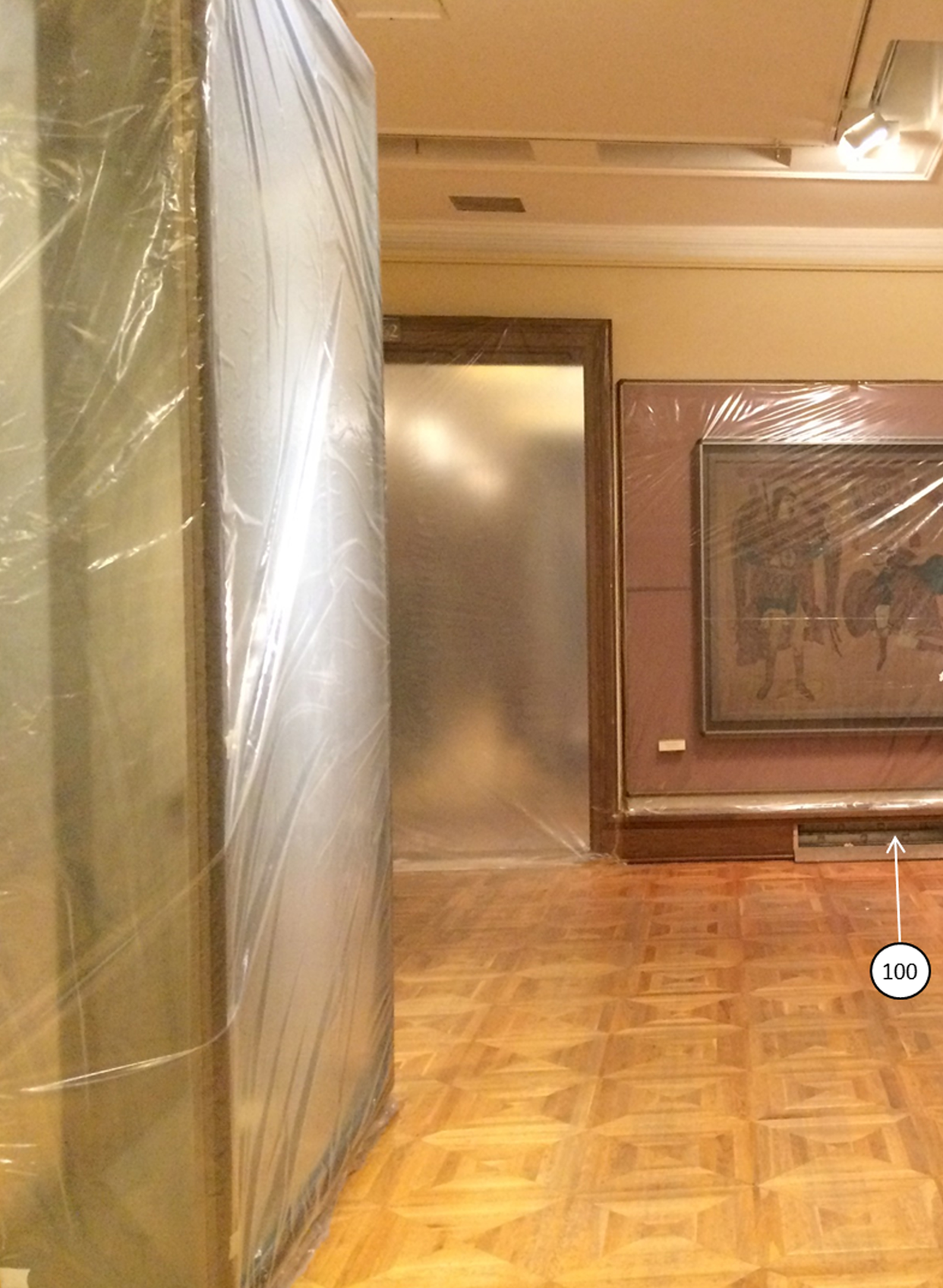

Supplement: S2 Fig — Arrow indicate site for sample; number in circle corresponds to the sample number (100). (TIF) [file pone.0230591.s002.tif]

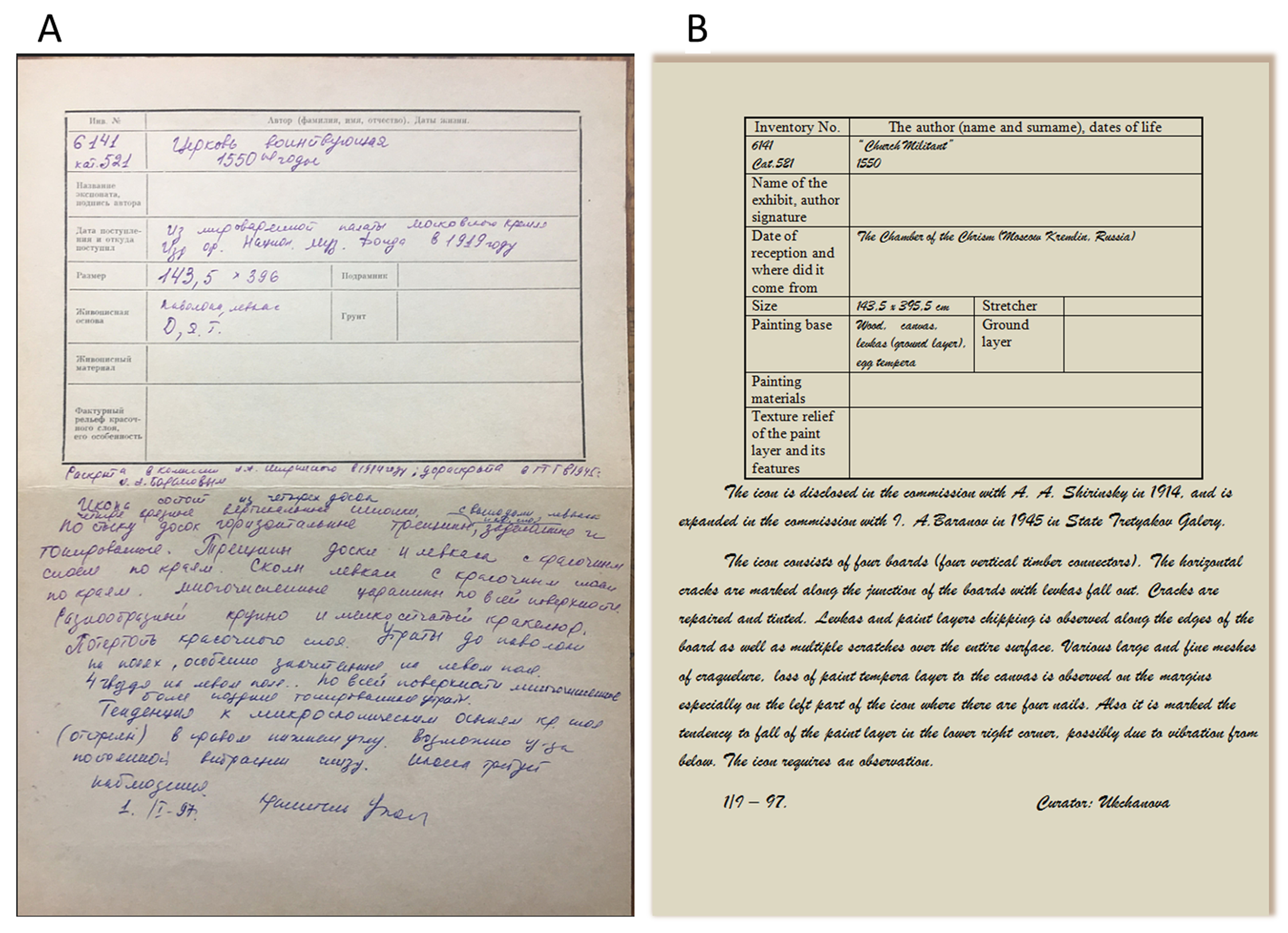

Supplement: S3 Fig — (A) Scan from the original document from the archive of STG (text–in Russian). (B) English translation of the original passport. (TIF) [file pone.0230591.s003.tif]

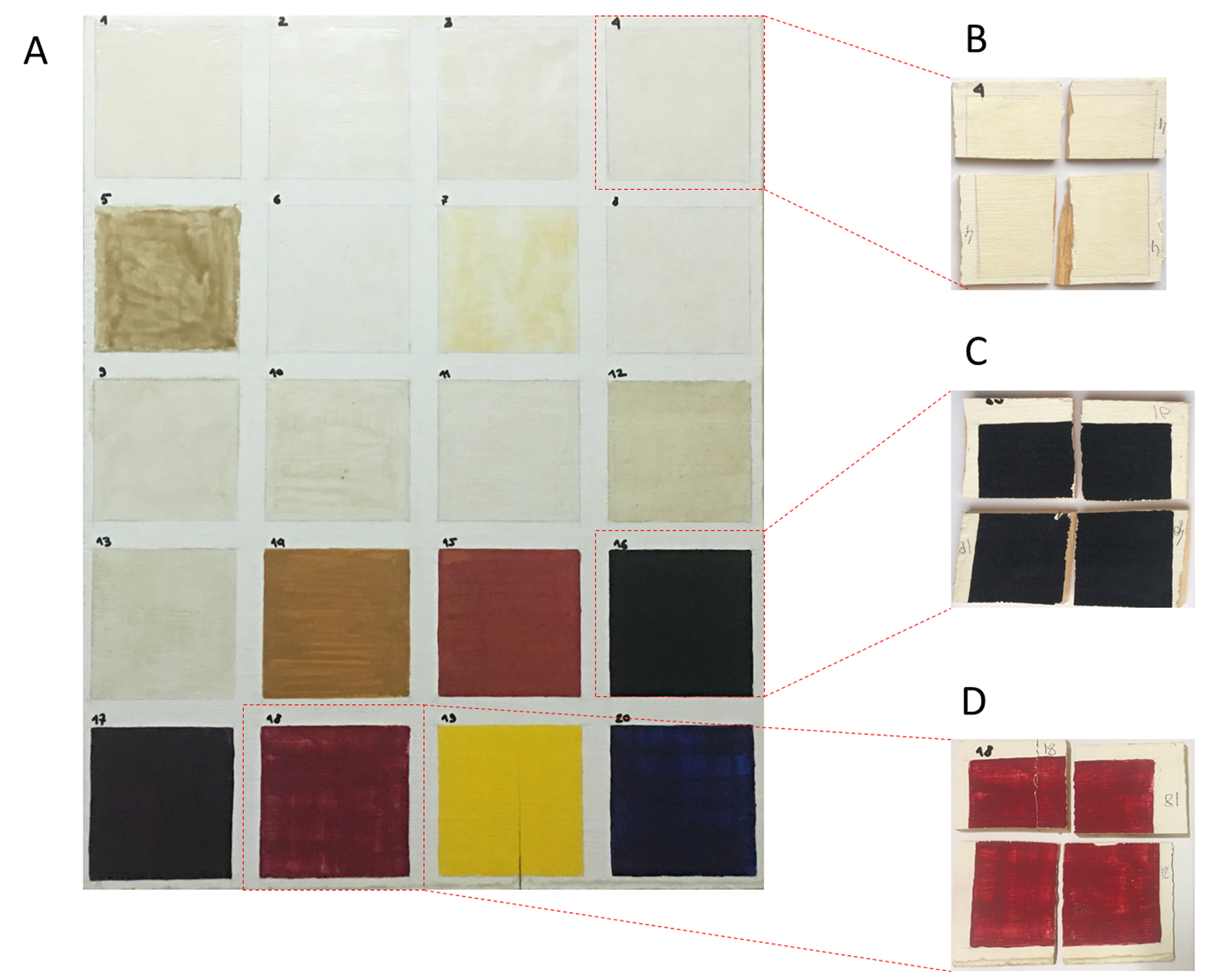

Supplement: S4 Fig — (A) Original desk with applied mock layers. (B-D) Defragmented mock layers: (B) mock layer 4; (C) mock layer 16; (D) mock layer 18. (TIF) [file pone.0230591.s004.tif]

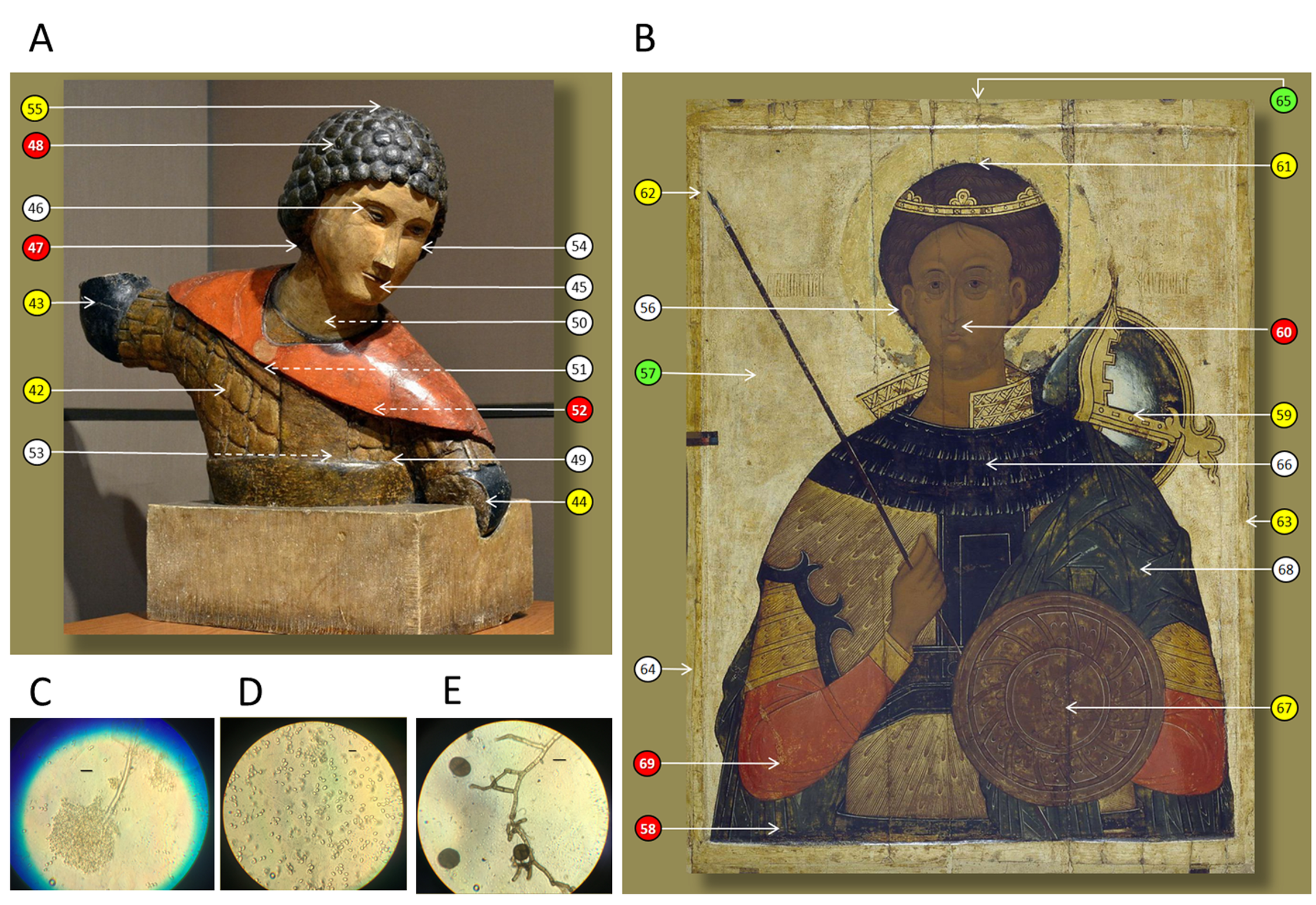

Supplement: S5 Fig — Samples distribution with: (A) bust of the “Saint George the Victorious”, (B) icon “Great Martyr St. Demetrius of Thessalonica”. Light microscopy analysis after sampling from objects I-III, scale bar = 10 μm (the species identity was determined after genotyping): (C) Aspergillus creber, (D) Mucor circinelloides, (E) Ulocladium chartarum. Arrows with solid lines correspond for sampling from tempera (front) side; arrows with doted lines–for sampling from rear side. Numbers in circles correspond to sample numbers: 42–55 –object II; 56–69 –object III. Cultivation on LB and CD media: yellow circles–growing only on LB medium, green circles–growing only on CD, red circles–growing on LB and CD, white circles–no growth on both mediums. (TIF) [file pone.0230591.s005.tif]

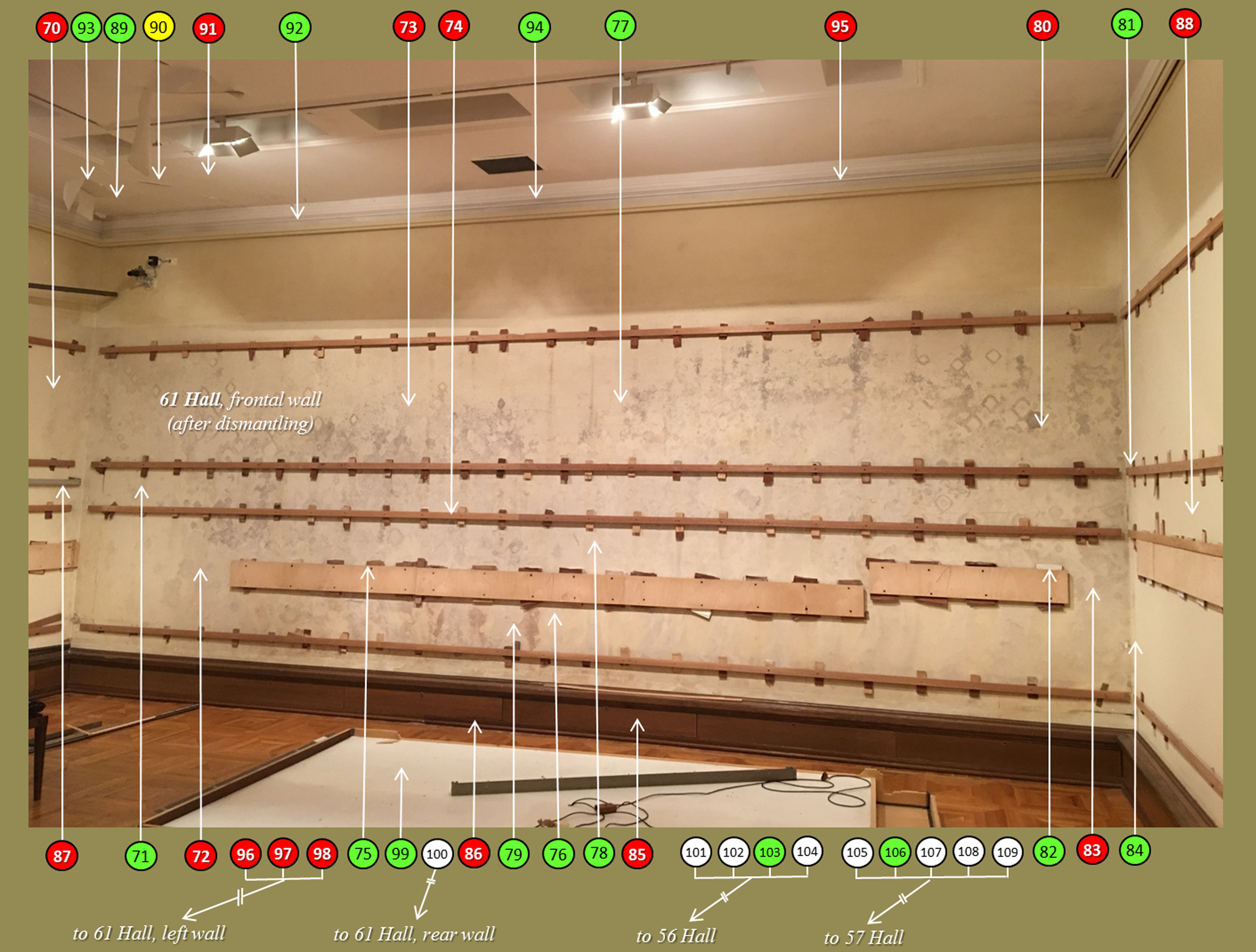

Supplement: S6 Fig — Arrows indicate sampling sites. Numbers in circles correspond to the sample numbers: 70–99 –Hall №61, frontal wall and nearby; 100 –Hall 61, rear wall; 101–104 –Hall 56; 105–109 –Hall 57. Cultivation on LB and CD media: yellow circles–growing only on LB medium, green circles–growing only on CD, red circles–growing on LB and CD, white circles–no growth on both media. (TIF) [file pone.0230591.s006.tif]

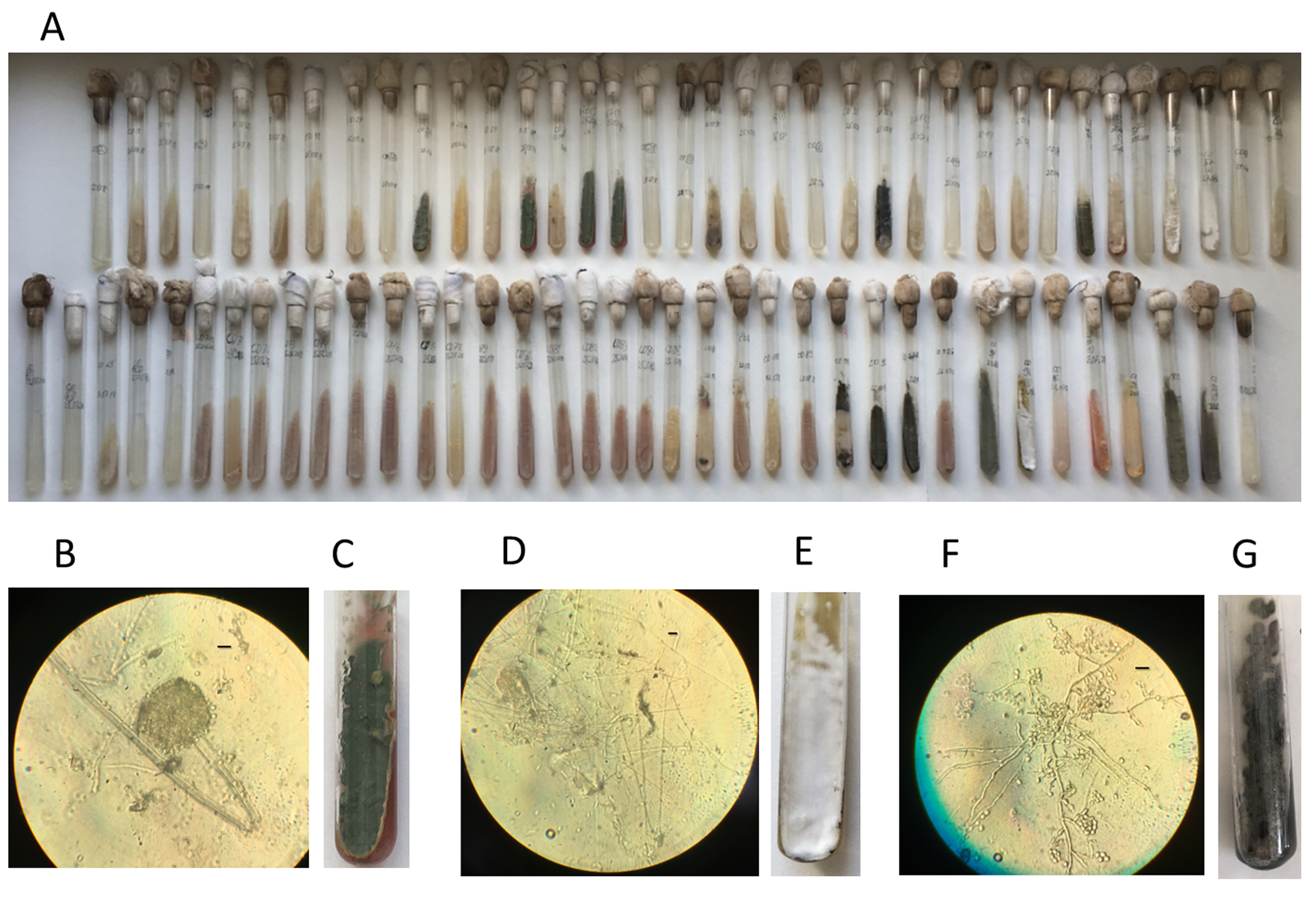

Supplement: S7 Fig — (A, C, E and G)–initial isolates growth on slant agar. (B, D and F)–light microscopy analysis, scale bar = 10 μm. Samples and corresponding species after genotyping: (B and C)–sample 27 (Aspergillus versicolor); (D and E)–sample 96 (Simplicillium lamellicola); (F and G)–sample 103 (Cladosporium cladosporioides). (TIF) [file pone.0230591.s007.tif]

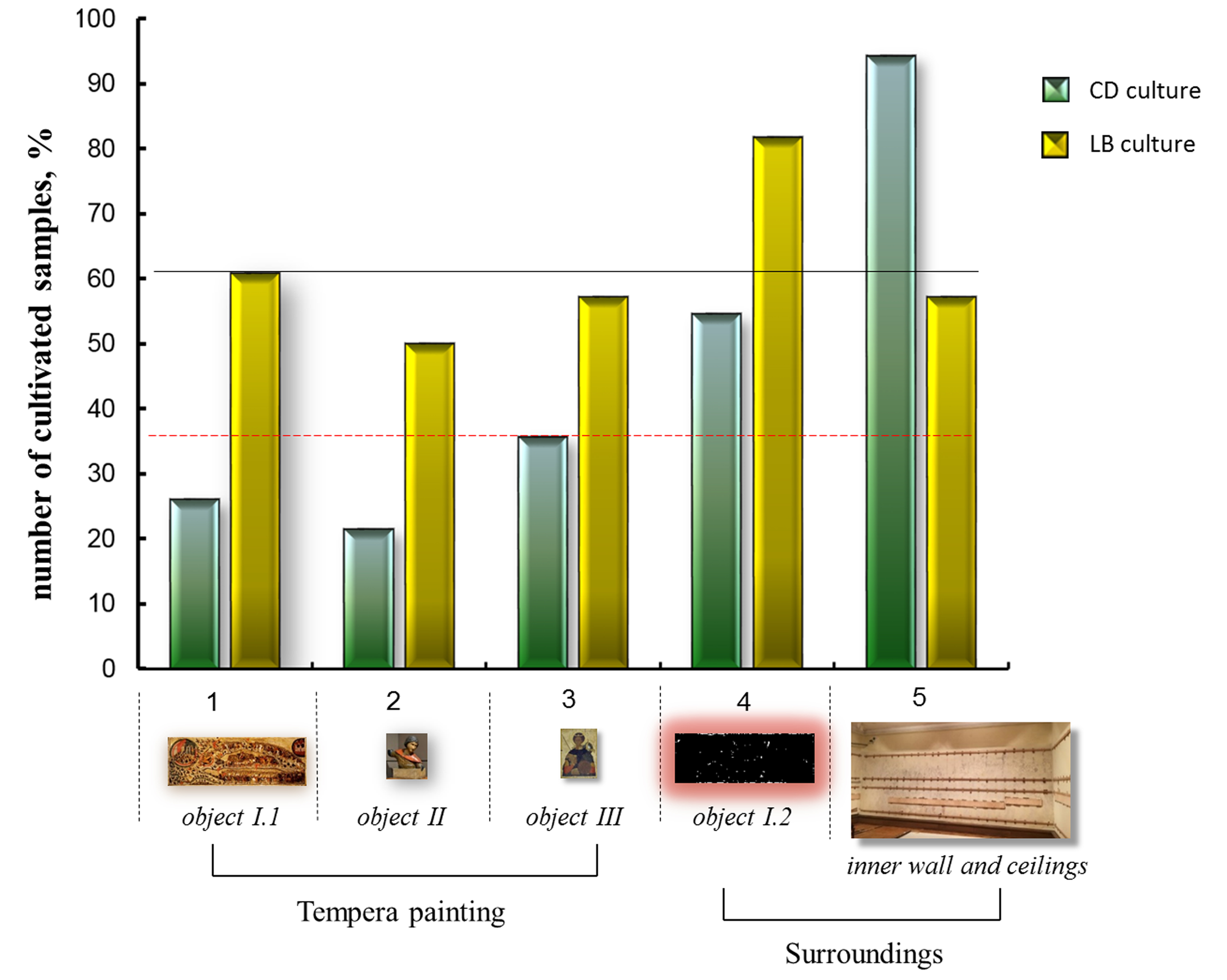

Supplement: S8 Fig — Samples growth from: 1 –object I, tempera side; 2 –object II; 3 –object III; 4 –object I, rear side and nearby; 5 –frontal wall (internal part) and ceilings. Solid line corresponds to maximum percent number of cultivable isolates, obtained from tempera painting objects, on LB medium; dotted line–to maximum number of cultivable isolates, obtained from tempera painting on CD medium. (TIF) [file pone.0230591.s008.tif]

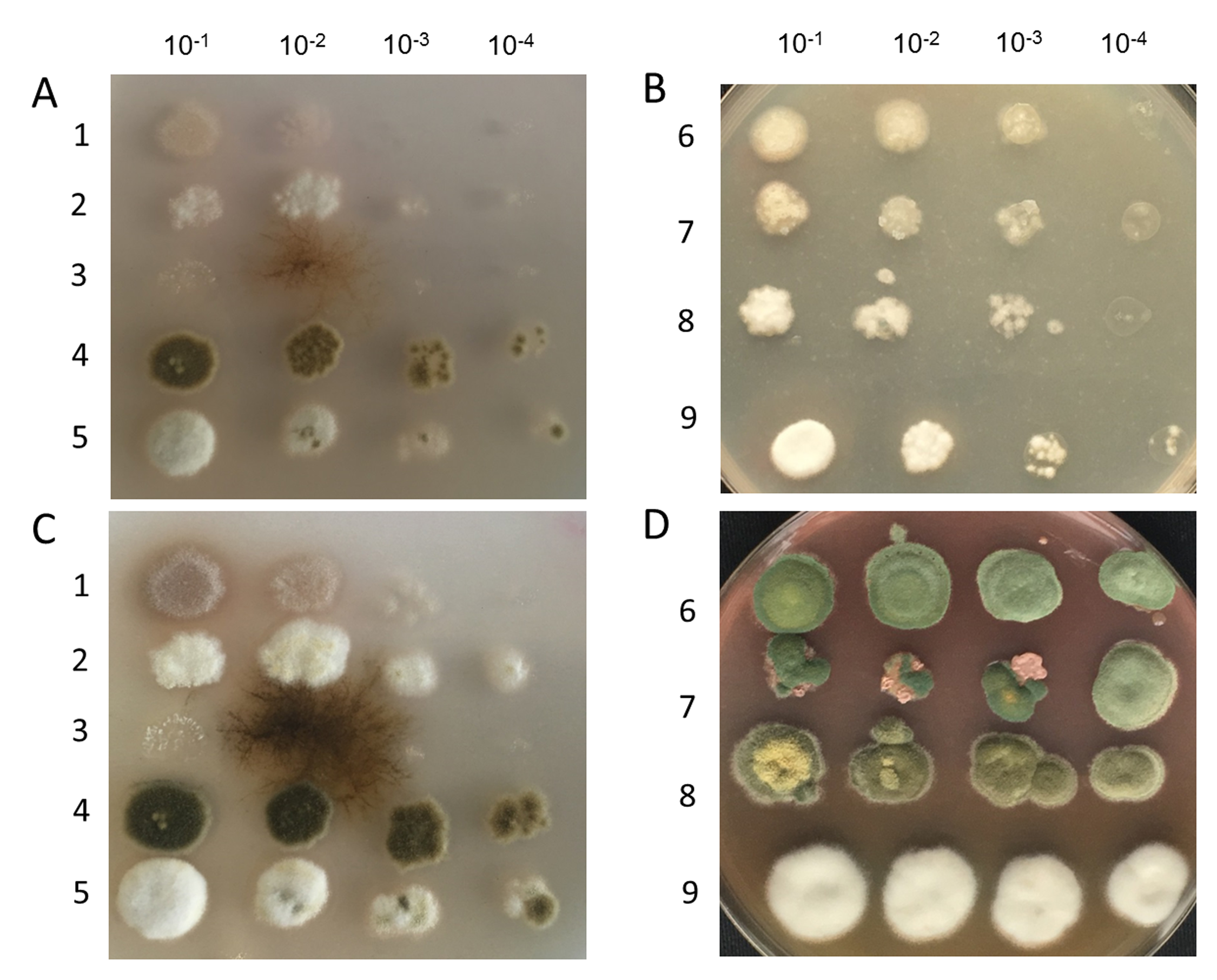

Supplement: S9 Fig — Drop and dilution assay, 25°C. (A and C) First set of isolates; (B and D) Second set of isolates. (A and B)– 3, (C)– 4, (D)– 12 days. Samples: 1–103; 2–106; 3–36; 4 – 93B; 5 – 93W; 6 – 25G; 7–86; 8–57; 9–96. (TIF) [file pone.0230591.s009.tif]

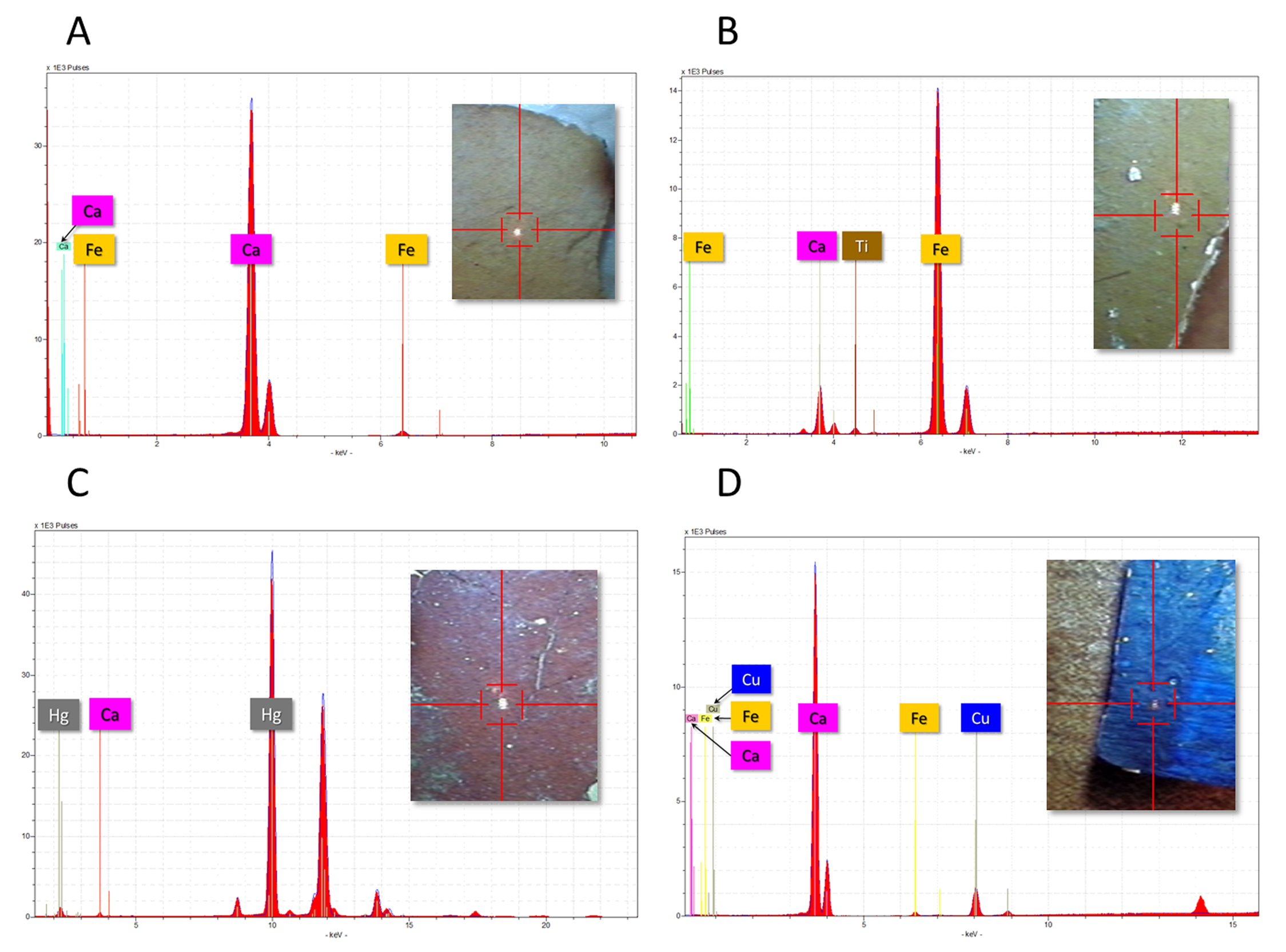

Supplement: S10 Fig — X-ray fluorescence spectra and probes for sampling (analyzing site is point into the center of red box). (A) 7 mock layer, (B) 14 mock layer, (C) 15 mock layer, (D) 20 mock layer. (TIF) [file pone.0230591.s010.tif]

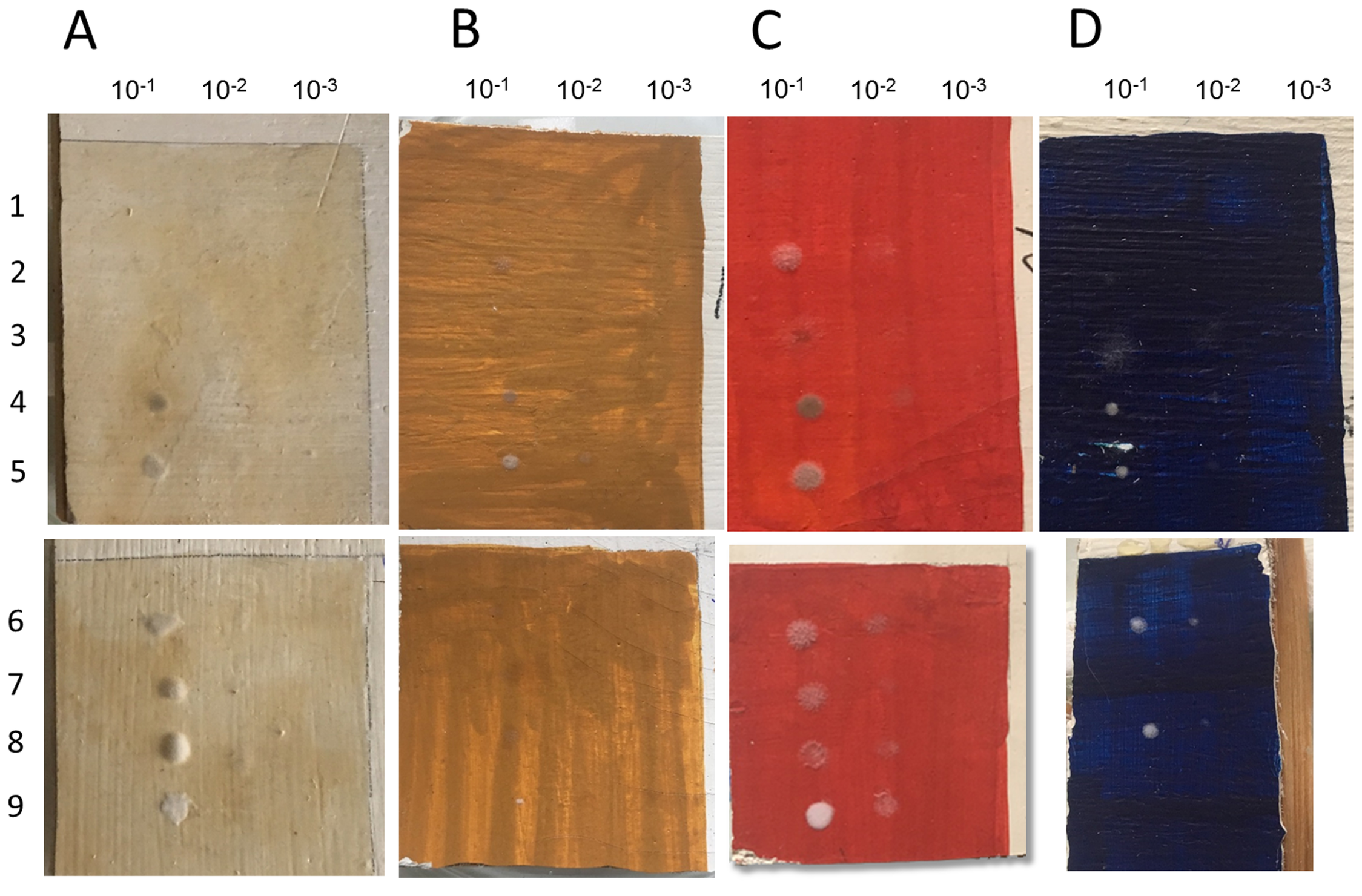

Supplement: S11 Fig — Drop and dilution assay, 48 h, 25°C. Mock layers: (A) 7 mock layer, (B) 14 mock layer, (C) 15 mock layer, (D) 20 mock layer. Samples: 1–103, 2–106, 3–36, 4 – 93B, 5 – 93W, 6 – 25G, 7–86, 8–57, 9–96. (TIF) [file pone.0230591.s011.tif]

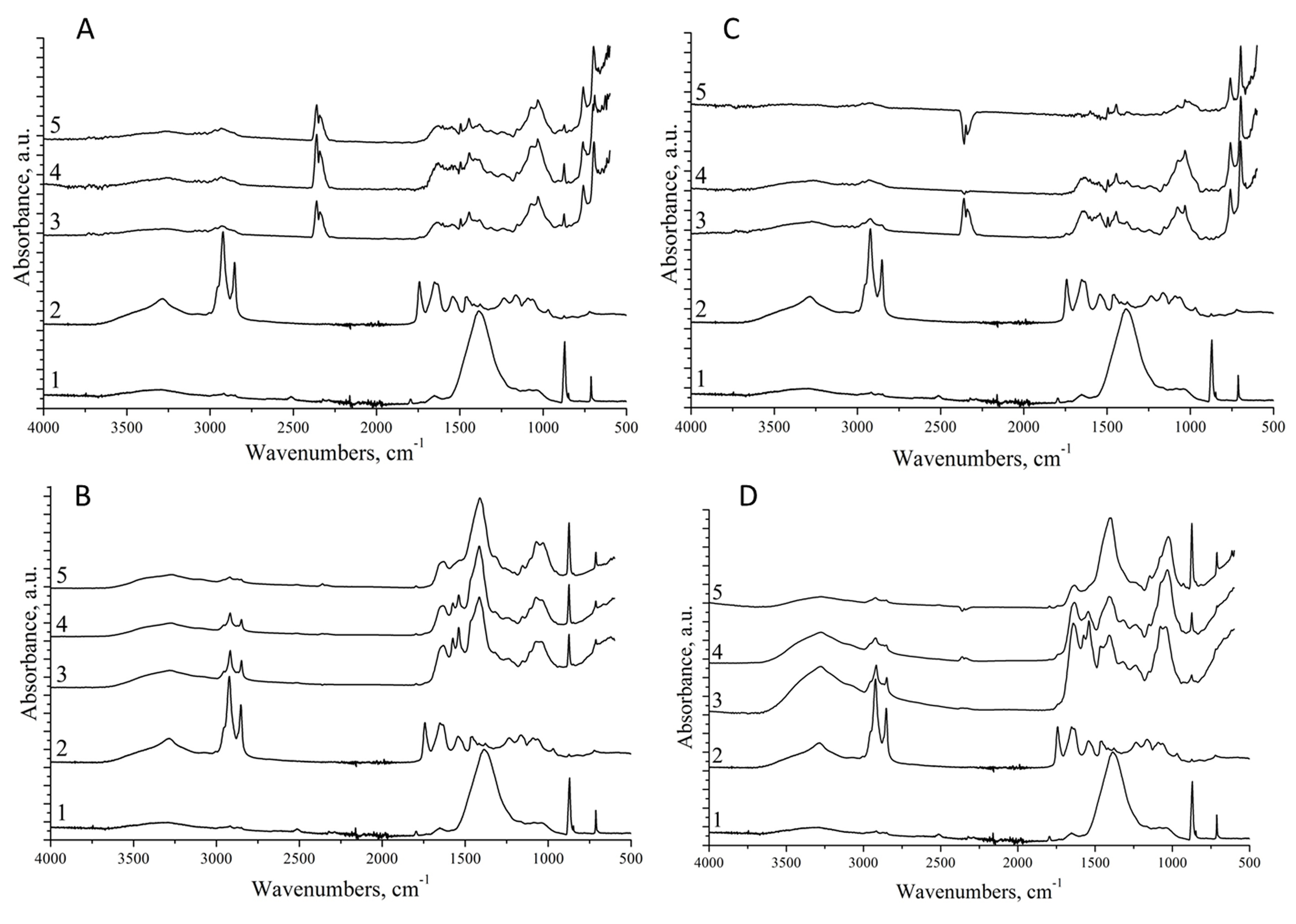

Supplement: S12 Fig — (A and B) Mock layer 7, inoculum–sample 36 (with fungus U. chartarum); (C and D) Mock layer 7, inoculum–sample 93W (with fungus A. creber). (A and C) Initial probes with microbe topsheet; (B and D) After washing from microbes. FTIR spectra: 1 –intact levkas, 2 –intact mock layer 15, 3 –periphery of inoculated zone; 4 –areola zone; 5 –center of inoculated zone. (TIF) [file pone.0230591.s012.tif]

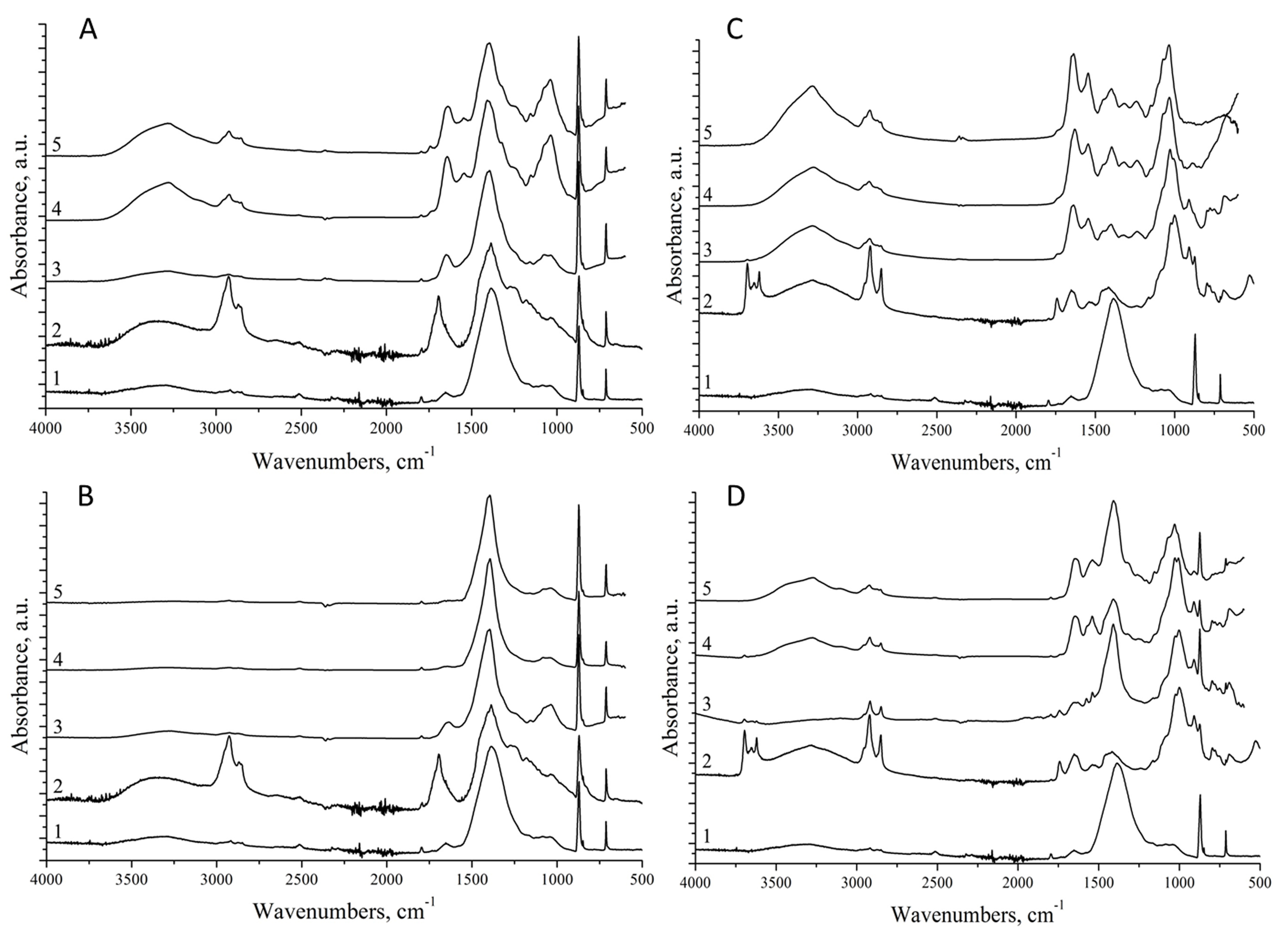

Supplement: S13 Fig — (A and B) Mock layer 9, inoculum–sample 93W (with fungus A. creber); (C and D) Mock layer 14, inoculum–sample 103 (with fungus C. cladosporioides). (A and C) Initial probes with microbe topsheet; (B and D) After washing from microbes. FTIR spectra: 1 –intact levkas, 2 –intact mock layer 15, 3 –periphery of inoculated zone; 4 –areola zone; 5 –center of inoculated zone. (TIF) [file pone.0230591.s013.tif]

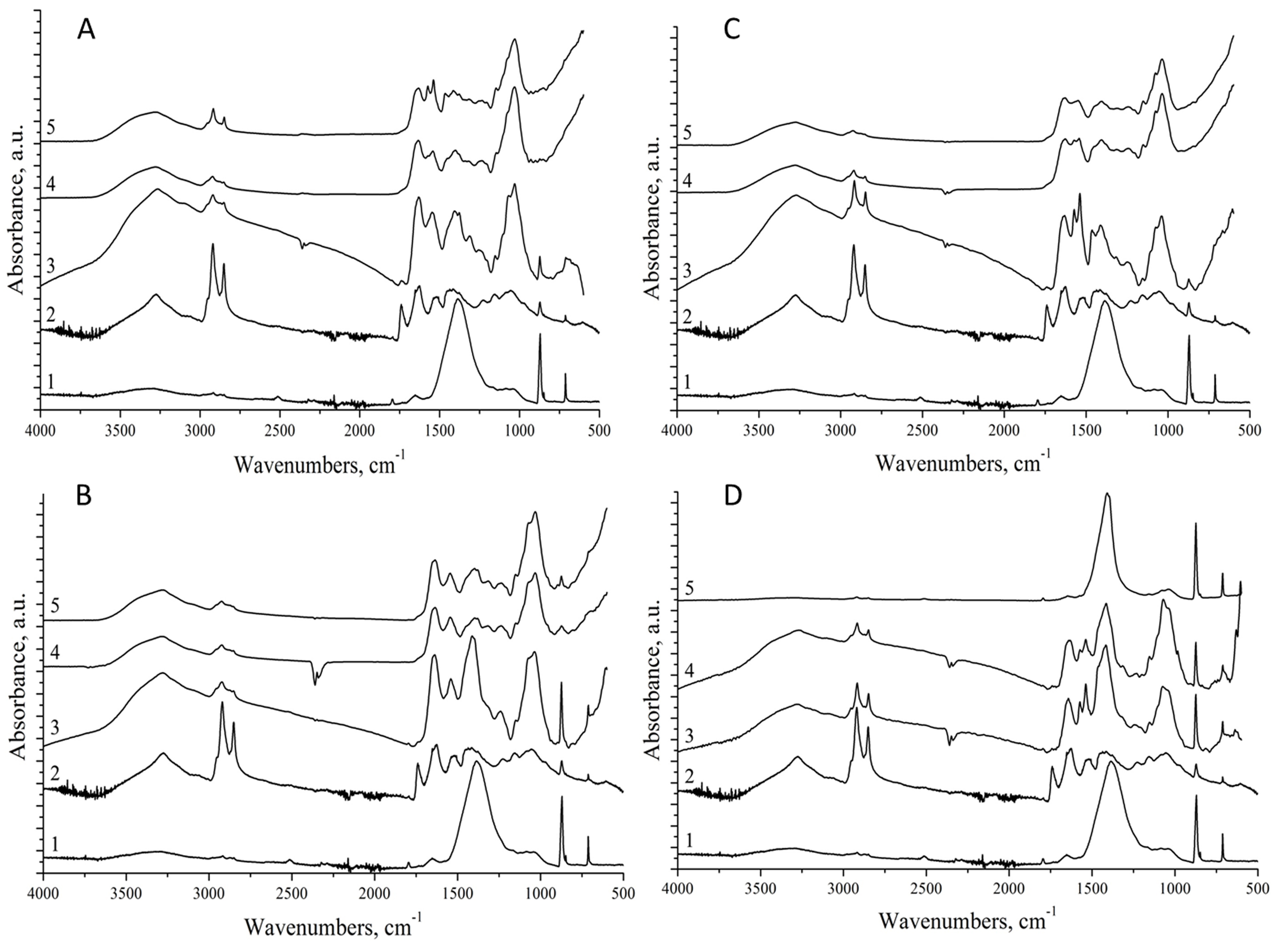

Supplement: S14 Fig — (A and B) Mock layer 15, inoculum–sample 106 (with fungus A. amoenus); (C and D) Mock layer 15, inoculum–sample 93B (with fungus C. parahalotolerans). (A and C) Initial probes with microbe topsheet; (B and D) After washing from microbes. FTIR spectra: 1 –intact levkas, 2 –intact mock layer 15, 3 –periphery of inoculated zone; 4 –areola zone; 5 –center of inoculated zone. (TIF) [file pone.0230591.s014.tif]

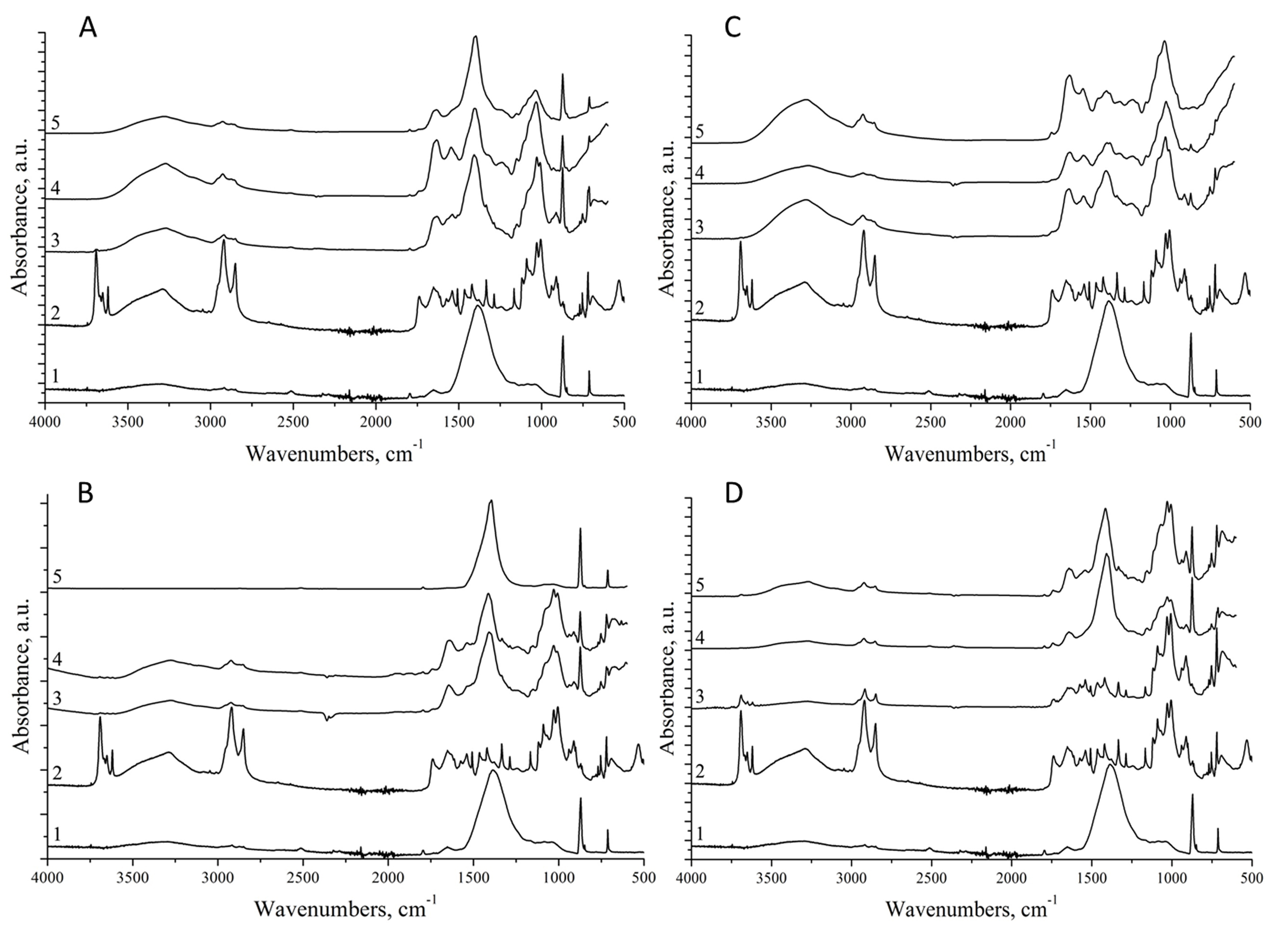

Supplement: S15 Fig — (A and B) Mock layer 20, inoculum–sample 93W (with fungus A. creber); (C and D) Mock layer 20, inoculum–sample 25G (with fungus A. versicolor). (A and C) Initial probes with microbe topsheet; (B and D) After washing from microbes. FTIR spectra: 1 –intact levkas, 2 –intact mock layer 15, 3 –periphery of inoculated zone; 4 –areola zone; 5 –center of inoculated zone. (TIF) [file pone.0230591.s015.tif]
