# Supplementary material for: Detection of potential biodeterioration risks for tempera painting in 16th century exhibits from State Tretyakov Gallery
Source: PLoS One. 2020 Apr 2;15(4):e0230591. doi: 10.1371/journal.pone.0230591 (PMC7117676; doi:10.1371/journal.pone.0230591)
Supplement: S1 Table — * Used materials applied over the levkas layer for mock layer preparation. (DOCX) [file pone.0230591.s016.docx]

**S1 Table. Materials, used for application of mock layers.**

| **Mock layer No.** | **Used materials^*^** | **Purpose** | **Manufacturer** |
| --- | --- | --- | --- |
| № 1 | Sturgeon glue | The binder in ground layer, restoration works | STG, restoration workshop |
| № 2 | Sturgeon glue, with SPCP (1%) | The binder in ground layer, restoration works | STG, restoration workshop |
| № 3 | Sturgeon glue SPCP (1%) and plasticizer honey (1%) | The binder in ground layer, restoration works | STG, restoration workshop |
| № 4 | Sturgeon glue with SPCP (1%) and plasticizer glycerol (3%) | The binder in ground layer, restoration works | STG, restoration workshop |
| № 5 | Joiner's glue | For boards gluing | STG, restoration workshop |
| № 6 | Gum arabic | Plasticizer | Merck, USA |
| № 7 | Egg emulsion (yolk and water) | The binder | STG restoration workshop, Russia |
| № 8 | Egg white | The binder | STG restoration workshop, Russia |
| № 9 | Rosin | Plasticizer, used as polymerization catalyst in oils | STG restoration workshop, Russia |
| № 10 | Wax-oil mastic | Neutral resin, covering layer in paints | STG restoration workshop, Russia |
| № 11 | Natural beeswax | Covering and protection | STG restoration workshop, Russia |
| № 12 | Linseed oil | Protection and binding | STG restoration workshop, Russia |
| № 13 | Dammar varnish | The coniferous resin | Ferrerio S.p.A., Italy |
| № 14 | Natural egg tempera (pigment - ochre, binder - yolk emulsion) | Yellow-brownish pigment of natural origin (iron oxide hydrate mixed with clay) | STG restoration workshop, Russia |
| № 15 | Natural egg tempera (pigment - cinnabar, binder - yolk emulsion) | Cinnabar pigment of mineral origin (mercury sulfide) | STG restoration workshop, Russia |
| № 16 | Natural egg tempera (pigment - boneblack, binder - yolk emulsion) | Black pigment of organic origin (burnt bone) | STG restoration workshop, Russia |
| № 17 | Egg manufacturing tempera – “Rowney” (Violet Alizarin) | Violet pigment of plant origin | Daler-Rowney Ltd, United Kingdom |
| № 18 | Egg manufacturing tempera – “Rowney” (Crimson Alizarin) | Crimson paint of plant origin | Daler-Rowney Ltd, United Kingdom |
| № 19 | Egg manufacturing tempera – “Rowney” (Cadmium Yellow) | Yellow pigment of chemical origin | Daler-Rowney Ltd, United Kingdom |
| № 20 | Egg manufacturing tempera – “Rowney” (Monestial Blue Phthalo) | Blue synthetic pigment (copper phthalocyanine) | Daler-Rowney Ltd, United Kingdom |

*^*^* Used materials applied over the levkas layer for mock layer preparation.
