# Supplementary material for: Detection of potential biodeterioration risks for tempera painting in 16th century exhibits from State Tretyakov Gallery
Source: PLoS One. 2020 Apr 2;15(4):e0230591. doi: 10.1371/journal.pone.0230591 (PMC7117676; doi:10.1371/journal.pone.0230591)
Supplement: S2 Table — (DOCX) [file pone.0230591.s017.docx]

**S2 Table. Cultured microorganisms used for inoculation of mock layers.**

| Set | Position in set | Original isolate number | Fungi,  dominant species | Prokaryote,  dominant species |
| --- | --- | --- | --- | --- |
| 1 | 1.1 | 103 | *Cladosporium cladosporioides* | *Stenotrophomonas sp* |
| 1 | 1.2 | 106 | *Aspergillus amoenus* | *Bacillus sp.* |
| 1 | 1.3 | 36 | *Ulocladium chartarum* | *Bacillus amyloliquefaciens* |
| 1 | 1.4 | 93B | *Cladosporium parahalotolerans* | *Achromobacter sp.* |
| 1 | 1.5 | 93W | *Aspergillus creber* | *Achromobacter sp.* |
| 2 | 2.1 | 25G | *Aspergillus versicolor* | *Stenotrophomonas maltophilia* |
| 2 | 2.2 | 86 | *Aspergillus versicolor* | *Bacillus amyloliquefaciens* |
| 2 | 2.3 | 57 | *Aspergillus creber* | *Brachybacterium sp.* |
| 2 | 2.4 | 96 | *Simplicillium lamellicola* | *Microbacterium sp.* |
