# Supplementary material for: Detection of potential biodeterioration risks for tempera painting in 16th century exhibits from State Tretyakov Gallery
Source: PLoS One. 2020 Apr 2;15(4):e0230591. doi: 10.1371/journal.pone.0230591 (PMC7117676; doi:10.1371/journal.pone.0230591)
Supplement: S3 Table — (DOCX) [file pone.0230591.s018.docx]

**S3 Table. Component composition of mock layers according XRF spectroscopy.**

| **Sample** | **Used materials** | **Detectable components** | | | |
| --- | --- | --- | --- | --- | --- |
|  |  | ***Levkas*** | | ***Tempera*** | |
|  |  | *Major* | *Trace* | *Major* | *Trace* |
| Levkas (ground layer) | *chalk, sturgeon glue* | Ca | Fe | - | - |
| Mock layer 7 | *Egg emulsion (yolk and water)* | Ca | Fe | - | - |
| Mock layer 14 | *Natural egg tempera (pigment - ochre, binder - yolk emulsion)* | Ca | Fe | Fe | K, Ti |
| Mock layer 15 | *Natural egg tempera (pigment - cinnabar, binder - yolk emulsion)* | Ca | Fe | Hg | Ba |
| Mock layer 20 | *Egg manufacturing tempera, “Rowney” (Monestial Blue Phthalo)* | Ca | Fe | Cu | - |
