# Supplementary material for: Detection of potential biodeterioration risks for tempera painting in 16th century exhibits from State Tretyakov Gallery
Source: PLoS One. 2020 Apr 2;15(4):e0230591. doi: 10.1371/journal.pone.0230591 (PMC7117676; doi:10.1371/journal.pone.0230591)
Supplement: S1 File — (DOCX) [file pone.0230591.s019.docx]

**S1 Materials and methods**

**A brief summary about objects of tempera painting for sampling, the 16^th^ century exhibits from the State Tretyakov Gallery**

**Object I. Grand Russian Orthodox icon “The Church Militant”, also known as, “Blessed are the warriors of the Celestine King”.**

Size of the Grand icon – 143.5 x 395.5 cm. The icon was written in the middle of the 16^th^ century (after 1552), the exact date is unknown. Material – tempera painting on wood. This icon is traditionally perceived as an allegorical representation of the conquest of the Kazan Khanate. It was commissioned by Tsar Ivan IV (Ivan the Terrible) in memory of his Kazan campaign of 1552. The icon was located at the southern gate of the Assumption Cathedral of the Moscow Kremlin near the Tsar's Place. At the beginning of the 20th century, the icon was in the Kremlin Chamber of Chrism, and in 1919 entered the collection of the State Tretyakov Gallery. The burning city, depicted in the icon, is Kazan; the city on the mountain – is Jerusalem of Heaven and Earth, which correlates, with Moscow. Warriors without nimbuses are the participants in the campaign; warriors with nimbuses are the Russian Holy princes Alexander Nevsky, Dmitry Donskoy and other Holy warriors. The rider with the cross is Vladimir Monomakh; behind him are three princes, Vladimir Svyatoslavovich and his two sons, Boris and Gleb. Young soldier with banner is Ivan the Terrible. The supposed author of the icon is considered to be the proto-priest of the Annunciation Cathedral of the Kremlin and the royal confessor Andrey.

**Object II. Limestone bust, covered in tempera, of the “Saint George the Victorious”.**

This bust represents the surviving fragment of full-sized statue. The limestone sculpture with tempera paintings of St. George the Victorious was installed on the Frolovsky gate (now, Spassky gate) of the Moscow Kremlin in 1464. Sculptor and architect is Vasily Yermolin - one of the most famous architects of Tsar Ivan III. During the Kremlin reconstruction, which involved Italian architects, the statue was carefully transferred to the Ascension Monastery, located next to the Frolovsky Gate. In the post-revolutionary time, the famous Ascension Monastery was demolished (1929); the upper part of the sculpture (bust part) was transferred to the State Tretyakov Gallery.

**Object III. Russian Orthodox icon “Great Martyr St. Demetrius of Thessalonica”.**

The grate Russian Orthodox icon, 96.5 x 122.5 cm, 16^th^ century, tempera painting on wood.

**Genomic DNA isolation.**

For genomic DNA isolation the initial samples were lyophilized. The cultivated microbial isolates were taken from slant agar media by bacteriological loop, suspended in 200 μl of Н_2_О to OD_600_ = 5–10 and lyophilized. Lyophilized samples resuspended in 200 μl of TES buffer (1% SDS, 1 mM EDTA, 100 mM Tris-HCl, pH 8.5), an equal volume of glass beads (D = 500 μm) and 200 μl of phenol (saturated 0.2 M Tris-HCl, pH 8.5) The mixture was vigorously shaken on a Vortex for 5 min, incubated for 65°C for 1 h, shaken for 5 min on Vortex, centrifuged for 10 min at 13.400 rpm on a MiniSpin apparatus (Eppendorf, Germany). Then the upper aqueous fraction was collected, washed with an equal volume of chloroform/ isoamyl alcohol (24:1), shaken for 1 min, incubated for 5 min at room temperature, shaken for 1 min, centrifuged 5 min at 13400 rpm. To the upper aqueous fraction 1/10 volume of 3M potassium acetate (pH = 5.2) and 3 volumes of 96% chilled ethyl alcohol were added. The solution was incubated 20 min, -20°C, centrifuged 13.400 rpm, the precipitate was washed with chilled 70% ethanol, dried, and resuspended in 20 – 30 μl of H_2_O. The resulting samples with genomic DNA of microorganisms were stored at -20°C.
