# Supplementary material for: Detection of potential biodeterioration risks for tempera painting in 16th century exhibits from State Tretyakov Gallery
Source: PLoS One. 2020 Apr 2;15(4):e0230591. doi: 10.1371/journal.pone.0230591 (PMC7117676; doi:10.1371/journal.pone.0230591)
Supplement: S2 File — (DOCX) [file pone.0230591.s020.docx]

**S2 Results and discussion**

**The characteristic of mock layers by XRF spectroscopy**

Non-destructive elemental analysis, performed by XRF spectroscopy, revealed a characteristic composition of different mock layers. The power of this method enables to detect elements with periodic number of 20 or higher by combined screening of several applied layers (due to the penetrating power of X-rays). Thus, when screening mock layers, which are the layering of various materials on levkas, the components of the levkas and the applied material are determined simultaneously. It is important to notice that major components in living organisms are not detected by XRF spectroscopy due to the limitations of the method; microelements are not detected due to the sensitivity of the method. Thus, in the case of surface growth of microorganisms it is possible to study the biodegradation of investigated materials even without washing from microbial layer. Such removal is required for micro-FTIR spectroscopy purposes; the layer with damaging microbiome did not allow for used long-wave radiation to scan materials condition in bottom layers).

**The analysis of micro-FTIR data of mock layers with biodeterioration**

IR-spectroscopy results of the initial rosin-based samples (S13 A, B Fig) confirmed the presence of specified material. Moreover, in the case of unwashed from microbial layer probe, characteristic for *Aspergillus* absorption bands from vibrations of polysaccharide (1200-900 cm^-1^) and protein (1700-1500 cm^-1^) functional groups were detected. These signals increased from periphery to the inoculation zone. At the same time, signals from calcium carbonate (levkas component) ware observed in all areas, which may indicate on small thickness of both the rosin and microbes layers. Washing from microorganisms revealed some rosin degradation. Fungal bands were not detected; this may mean they did not spread into the depth of levkas, perhaps due to more dense structure of rosin (as well as other resins and varnishes) in comparison with sturgeon glue or egg tempera (proteins).

The set of absorption bands in the spectrum of the original sample corresponds to the literature data indicating the content of ochre as a yellow pigment (S13 C, D Fig). Difficulties in decoding arise due to the fact that the main absorption band (1200-900 cm^-1^) from this pigment intersects with the signals from the polysaccharides that make up the fungi (*C. cladosporioides*). However, a decrease in the intensity of other relevant ochre absorption bands (in particular, with maximum at 910 cm^–1^) in the spectra from the periphery to the center confirms the increase in the quantum of *C. cladosporioides*.

Due to the mercuric sulphide characteristic bands are behind the investigation limits of wave-number scale (the maximums are at 270 and 330 cm^-1^) FTIR-spectroscopy results do not allowed to estimate the presence of cinnabar pigment in mock layer 15 (S14 C, D Fig). At the same time, an increase in the intensity of the absorption bands characteristic of polysaccharides is observed on the surface of the sample with the grown isolate from the periphery towards the center, which indicates an increase fungi quantum in this direction. According spectral data after removal of *C. parahalotolerans* by washing in the probe in the inoculated zone, the colored layer is removed completely (to the ground layer, levkas), which may be due to the more intensive interaction of the fungi deep down the material (S14 D*5* Fig). At the same time, a certain number of fungi remain on the periphery and in the intermediate region (areola zone), which is manifested in the presence of an intense absorption band in the range of wave numbers 1200–900 cm^–1^ (S14 D*4* Fig).

According FTIR-spectroscopy data the presence of CuFc is clearly registered (S15 C, D Fig) in egg manufacturing tempera “Rowney” samples (Monestial Blue Phthalo) which correlates with XRF spectroscopy data (S10 D Fig and S3 Table). At the same time, the behavior of fungi for these samples is close to the samples, based on natural egg tempera (S13.C, D and S14 A–D Figs). After washing from visual amounts of microorganisms (with *A. versicolor*) they remain in certain amount in all studied areas of the sample. In the case of inoculum with *A. creber* (sample 93W) more intensive interaction with tempera paint registered in the inoculated zone, since after its removal, the surface along the fungi is cleaned to the ground, where a noticeable quantum of fungi has not been fixed (S15 B*5* Fig).
